# Plant growth-promoting bacteria as inoculants in agricultural soils

**DOI:** 10.1590/S1415-475738420150053

**Published:** 2015

**Authors:** Rocheli de Souza, Adriana Ambrosini, Luciane M.P. Passaglia

**Affiliations:** Departamento de Genética, Instituto de Biociências, Universidade Federal do Rio Grande do Sul, Porto Alegre, RS, Brazil

**Keywords:** nitrogen fixation, siderophore production, plant-bacteria interaction, inoculant, rhizosphere

## Abstract

Plant-microbe interactions in the rhizosphere are the determinants of plant health, productivity and soil fertility. Plant growth-promoting bacteria (PGPB) are bacteria that can enhance plant growth and protect plants from disease and abiotic stresses through a wide variety of mechanisms; those that establish close associations with plants, such as the endophytes, could be more successful in plant growth promotion. Several important bacterial characteristics, such as biological nitrogen fixation, phosphate solubilization, ACC deaminase activity, and production of siderophores and phytohormones, can be assessed as plant growth promotion (PGP) traits. Bacterial inoculants can contribute to increase agronomic efficiency by reducing production costs and environmental pollution, once the use of chemical fertilizers can be reduced or eliminated if the inoculants are efficient. For bacterial inoculants to obtain success in improving plant growth and productivity, several processes involved can influence the efficiency of inoculation, as for example the exudation by plant roots, the bacterial colonization in the roots, and soil health. This review presents an overview of the importance of soil-plant-microbe interactions to the development of efficient inoculants, once PGPB are extensively studied microorganisms, representing a very diverse group of easily accessible beneficial bacteria.

## Introduction

**Figure 1:**
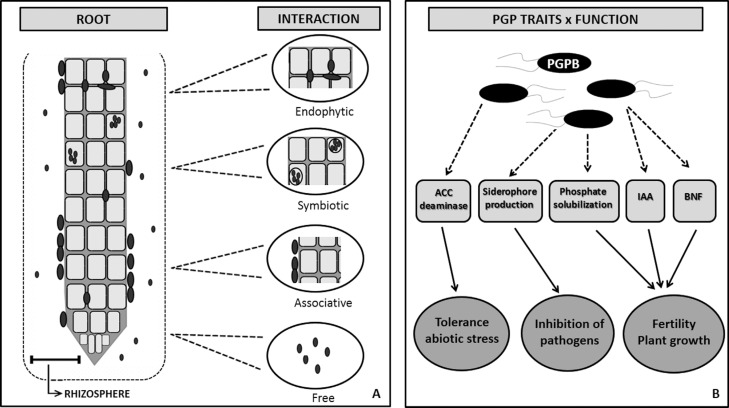
Rhizosphere/bacteria interactions. A) Different types of association between plant roots and beneficial soil bacteria; B) After colonization or association with roots and/or rhizosphere, bacteria can benefit the plant by (i) tolerance toward abiotic stress through action of ACC deaminase; (ii) defense against pathogens by the presence of competitive traits such as siderophore production; (iii) increase of fertility and plant growth through biological nitrogen fixation (BNF), IAA (indole-3-acetic acid) production, and phosphate solubilization around roots.

The rhizosphere can be defined as the soil region where processes mediated by microorganisms are specifically influenced by the root system (Figure 1A). This area includes the soil connected to the plant roots and often extends a few millimeters off the root surface, being an important environment for the plant and microorganism interactions (Lynch, 1990; Gray and Smith, 2005), because plant roots release a wide range of compounds involved in attracting organisms which may be beneficial, neutral or detrimental to plants (Lynch, 1990; Badri and Vivanco, 2009). The plant growth-promoting bacteria (or PGPB) belong to a beneficial and heterogeneous group of microorganisms that can be found in the rhizosphere, on the root surface or associated to it, and are capable of enhancing the growth of plants and protecting them from disease and abiotic stresses (Dimkpa *et al*., 2009a; Grover *et al*., 2011; Glick, 2012). The mechanisms by which PGPB stimulate plant growth involve the availability of nutrients originating from genetic processes, such as biological nitrogen fixation and phosphate solubilization, stress alleviation through the modulation of ACC deaminase expression, and production of phytohormones and siderophores, among several others.

Interactions between plants and bacteria occur through symbiotic, endophytic or associative processes with distinct degrees of proximity with the roots and surrounding soil (Figure 1A). Endophytic PGPB are good inoculant candidates, because they colonize roots and create a favorable environment for development and function. Non-symbiotic endophytic relationships occur within the intercellular spaces of plant tissues, which contain high levels of carbohydrates, amino acids, and inorganic nutrients (Bacon and Hinton, 2006).

Agricultural production currently depends on the large-scale use of chemical fertilizers (Wartiainen *et al*., 2008; Adesemoye *et al*., 2009). These fertilizers have become essential components of modern agriculture because they provide essential plant nutrients such as nitrogen, phosphorus and potassium. However, the overuse of fertilizers can cause unanticipated environmental impacts (Shenoy *et al*., 2001; Adesemoye *et al*., 2009). To achieve maximum benefits in terms of fertilizer savings and better growth, the PGPB-based inoculation technology should be utilized along with appropriate levels of fertilization. Moreover, the use of efficient inoculants can be considered an important strategy for sustainable management and for reducing environmental problems by decreasing the use of chemical fertilizers (Alves *et al*., 2004; Adesemoye *et al*., 2009; Hungria *et al*., 2010, 2013).

The success and efficiency of PGPB as inoculants for agricultural crops are influenced by various factors, among which the ability of these bacteria to colonize plant roots, the exudation by plant roots and the soil health. The root colonization efficiency of PGPB is closely associated with microbial competition and survival in the soil, as well as with the modulation of the expression of several genes and cell-cell communication via quorum sensing (Danhorn and Fuqua, 2007; Meneses *et al*., 2011; Alquéres *et al*., 2013; Beauregard *et al*., 2013). Plant roots react to different environmental conditions through the secretion of a wide range of compounds which interfere with the plant-bacteria interaction, being considered an important factor in the efficiency of the inoculants (Bais *et al*., 2006; Cai *et al*., 2009, 2012; Carvalhais *et al*., 2013). Soil health is another important factor that affects the inoculation efficiency, due to several characteristics such as soil type, nutrient pool and toxic metal concentrations, soil moisture, microbial diversity, and soil disturbances caused by management practices.

## Mechanisms of Plant Growth Promotion

The mechanisms by which bacteria can influence plant growth differ among species and strains, so typically there is no single mechanism for promoting plant growth. Studies have been conducted regarding the abilities of various bacteria to promote plant growth, among them the endophytic bacteria. Endophytes are conventionally defined as bacteria or fungi that colonize internal plant tissues, can be isolated from the plant after surface disinfection and cause no negative effects on plant growth (Gaiero *et al*., 2013). Many bacteria promote plant growth at various stages of the host plant life cycle through different mechanisms (Figure 1B). Here we discuss five important mechanisms of PGPB.

## Biological nitrogen fixation

All organisms require nitrogen (N) to synthesize biomolecules such as proteins and nucleic acids. However, the main source of N in nature, the atmospheric nitrogen (N_2_), is not accessible to most living organisms, including eukaryotes. Biological nitrogen fixation (BNF) is the process responsible for the reduction of N_2_ to ammonia (NH_3_) (Newton, 2000; Franche *et al*., 2009) and is performed in diazotrophic microorganisms, particularly bacteria and archaea (Dixon and Kahn, 2004).

Diazotrophic microorganisms perform BNF through nitrogenase, a highly conserved enzyme that comprises two metalloproteins, FeMo-protein and Fe-protein (Dixon and Kahn, 2004). Although there are many morphological, physiological and genetic differences between the diazotrophics, as well as an enormous variability of environments where they can be found, they all contain the enzyme nitrogenase (Moat and Foster, 1995; Boucher *et al*., 2003; Zehr *et al*., 2003; Dixon and Kahn, 2004). *Klebsiella oxytoca* M5a1 was the first diazotrophic bacterium that had the genes involved in the synthesis and functioning of nitrogenase (*nif*, N_2_ fixation) identified and characterized. In the genome of this bacterium, 20 *nif* genes are grouped in a 24-kb chromosomal region, organized into 8 operons: *nifJ, nifHDKTY, nifENX, nifUSVWZ, nifM, nifF, nifLA*, and *nifBQ* (Arnold *et al*., 1988). The *nifD* and *nifK* genes encode the FeMo-protein, and *nifH* encodes the Fe-protein (Boucher *et al*., 2003).

Among the leguminous plants of the Fabaceae family, the soil bacteria of the Rhizobiaceae (rhizobia) family are confined to the root nodules (Willems, 2007). Within these nodules, rhizobia effectively perform BNF through the adequate control of the presence of oxygen, an inhibitor of nitrogenase activity (Dixon and Kahn, 2004; Shridhar, 2012). Many species of microorganisms are used in the cultivation of plants of economic interest, facilitating the host plant growth without the use of nitrogenous fertilizers. For instance, the production of soybean (*Glycine max* L.) in Brazil is an excellent example of the efficiency of BNF through the use of different strains of *Bradyrhizobium* sp., such as *B. japonicum* and *B. elkanii* (Alves *et al*., 2004; Torres *et al*., 2012). The importance of endophytic N_2_-fixing bacteria has also been the object of studies in non-leguminous plants such as sugarcane (*Saccharum officinarum* L.; Thaweenut *et al*., 2011). Other studies have suggested that bradyrhizobia colonize and express *nifH* not only in the root nodules of leguminous plants but also in the roots of sweet potatoes (*Ipomoea batatas* L.), acting as diazotrophic endophytes (Terakado-Tonooka *et al*., 2008).

*Herbaspirillum* sp. is a gram-negative bacterium associated with important agricultural crops, such as rice (*Oryza sativa* L.; Pedrosa *et al*., 2011; Souza *et al*., 2013), sorghum (*Sorghum bicolor* L.*;* James *et al*., 1997), maize (*Zea mays* L.; Monteiro *et al*., 2008), and sugarcane (Pedrosa *et al*., 2011). Fluorescence and electron microscopy have revealed that the *Herbaspirillum* sp. strain B501 colonizes the intercellular spaces of wild rice (*Oryza officinalis*) leaves, fixing N_2_ *in vivo* (Elbeltagy *et al*., 2001), and expresses *nif* genes in wild rice shoots (You *et al*., 2005). This same strain also colonizes the intercellular spaces of the roots and stem tissues of sugarcane plants, showing non-specificity to the host plant (Njoloma *et al*., 2006). Inoculations of barley (*Hordeum vulgare* L.) and *Miscanthus* plants with a strain of *H. frisingense* showed that this bacterium is a true plant endophyte (Rothballer *et al*., 2008).

The PGPB related to genus *Azospirillum* have been largely studied because of their efficiency in promoting the growth of different plants of agronomical interest. Garcia de Salamone *et al*. (1996) showed that *Azospirillum* sp. contribute to plant fitness through BNF. The genus *Burkholderia* also includes species that fix N_2_. *B. vietnamiensis*, a human pathogenic species, was efficient in colonizing rice roots and fixing N_2_ (Govindarajan *et al*., 2008). In addition to *Burkholderia*, the potential of BNF and endophytic colonization of bacteria belonging to the genera *Pantoea, Bacillus* and *Klebsiella* was also confirmed in different maize genotypes (Ikeda *et al*., 2013). *Gluconacetobacter diazotrophicus* is another well-studied endophyte (Baldani *et al*., 1997; Oliveira *et al*., 2002; Muthukumarasamy *et al*., 2005; Bertalan *et al*., 2009). In conditions of N deficiency, the *G. diazotrophicus* strain Pal5 isolated from sugarcane is able to increase the N content when compared with plants inoculated with a Nif^−^ mutant or uninoculated plants (Sevilla *et al*., 2001).

## Production of indolic compounds

The influence of bacteria in the rhizosphere of plants is largely due to the production of auxin phytohormones (Spaepen *et al*., 2007). Several bacterial species can produce indolic compounds (ICs) such as the auxin phytohormone indole-3-acetic acid (IAA), which present great physiological relevance for bacteria-plant interactions, varying from pathogenesis to phytostimulation (Spaepen *et al*., 2007). The ability to produce ICs is widely distributed among plant-associated bacteria. Souza *et al*. (2013) demonstrated that approximately 80% of bacteria isolated from the rhizosphere of rice produce ICs. Other studies have shown that rhizosphere bacteria produce more ICs than bulk soil bacteria (Khalid *et al*., 2004), and in a recent study Costa *et al*. (2014) showed that this effect was also observed in endophytic bacteria, demonstrating high IC production in the Enterobacteriaceae family (*Enterobacter, Escherichia, Grimontella, Klebsiella, Pantoea*, and *Rahnella*).

The synthesis of ICs in bacteria depends on the presence of precursors in root exudates. Among the various exudates, L-tryptophan has been identified as the main precursor for the route of IC biosynthesis in bacteria. The characterization of intermediate compounds has led to the identification of different pathways that use L-tryptophan as the main precursor. The different pathways of IAA synthesis in bacteria show a high degree of similarity with the IAA biosynthesis pathways in plants (Spaepen *et al*., 2007). Beneficial bacteria predominantly synthesize IAA via the indole-3-pyruvic acid pathway, an alternative pathway dependent on L-tryptophan. In phytopathogenic bacteria, IAA is produced from L-tryptophan via the indol-acetoamide pathway. In *A. brasilense*, at least three biosynthesis pathways have been described for the production of IAA: two L-tryptophan-dependent (indole-3-pyruvic acid and indole-acetoamide pathways) and one L-tryptophan-independent (Prinsen *et al*., 1993), with the indole-3-pyruvic acid pathway as the most important among them (Spaepen *et al*., 2008).

The potential of rhizobia to establish symbiosis with legumes has been well documented; however, studies have indicated the importance of IAA in nodulation events. The co-inoculation of beans (*Phaseolus vulgaris* L.; cultivar DOR364) with *A. brasilense* Sp245 and *Rhizobium etli* CNPAF512 yielded a greater number of nodules; however, the results obtained using a mutant strain of *Azospirillum* that produced only 10% of the IAA of the wild-type strain were not satisfactory, indicating the importance of bacterial IAA in the establishment and efficiency of symbiosis (Remans *et al*., 2008). IAA-producing *Azospirillum* sp. also promoted alterations in the growth and development of wheat (*Triticum aestivum* L.) plants (Dobbelaere *et al*., 1999; Akbari *et al*., 2007; Spaepen *et al*., 2008; Baudoin *et al*., 2010).

Soil microorganisms are capable of synthesizing and catabolizing IAA. The capacity of catabolizing IAA has been well characterized in *B. japonicum* (Jensen *et al*., 1995) and *Pseudomonas putida* 1290 (Leveau and Lindow, 2005). *P. putida* 1290 uses IAA as the sole source of carbon (C), nitrogen (N), and energy. In addition to the utilization of IAA, strain 1290 also produced IAA in culture medium supplemented with L-tryptophan. In co-inoculation experiments in radish (*Raphanus sativus* L.) roots, this strain minimized the negative effects of high IAA concentrations produced by the pathogenic bacteria *Rahnella aquaticus* and *P. syringae*. In this context, microorganisms that catabolize IAA might also positively affect the growth of plants and prevent pathogen attack (Leveau and Lindow, 2005).

## Siderophore production

Iron (Fe) is an essential micronutrient for plants and microorganisms, as it is involved in various important biological processes, such as photosynthesis, respiration, chlorophyll biosynthesis (Kobayashi and Nishizawa, 2012), and BNF (Dixon and Kahn, 2004). In anaerobic and acidic soils, such as flooded soils, high concentrations of ferrous (Fe^2+^) ions generated through the reduction of ferric (Fe3^+^) ions might lead to iron toxicity due to excessive Fe uptake (Stein *et al*., 2009). Under aerobic conditions, iron solubility is low, reflecting the predominance of Fe^3+^ typically observed as oxyhydroxide polymers, thereby limiting the Fe supply for different forms of life, particularly in calcareous soils (Andrews *et al*., 2003; Lemanceau *et al*., 2009). Microorganisms have developed active strategies for Fe uptake. Bacteria can overcome the nutritional Fe limitation by using chelator agents called siderophores. Siderophores are defined as low-molecular-mass molecules (< 1000 Da) with high specificity and affinity for chelating or binding Fe^3+^, followed by the transportation and deposition of Fe within bacterial cells (Neilands, 1995; Krewulak and Vogel, 2008).

Various studies have shown that siderophores are largely produced by bacterial strains associated with plants. This characteristic was the most common trait found in isolates associated with sunflower (*Helianthus annuus* L.; Ambrosini *et al*., 2012) and rice (Souza *et al*., 2013). Notably, in rice roots, isolates belonging to genera *Enterobacter* and *Burkholderia* produced the highest levels of siderophores (Souza *et al*., 2013, 2014). Costa *et al*. (2014) simultaneously analyzed the PGPB datasets from seven independent studies that employed similar methodologies for bioprospection and observed that 64% of all isolates and 100% of all bacterial genera presented siderophore-producing strains. The bacterial genera *Burkholderia, Enterobacter* and *Grimontella* presented strains with high siderophore production, while the genera *Klebsiella, Stenotrophomonas, Rhizobium, Herbaspirillum* and *Citrobacter* presented strains with low siderophore production.

The excretion of siderophores by bacteria might stimulate plant growth, thereby improving nutrition (direct effect) or inhibiting the establishment of phytopathogens (indirect effect) through the sequestration of Fe from the environment. Unlike microbial pathogens, plants are not affected by bacterial-mediated Fe depletion, and some plants can even capture and utilize Fe^3+^-siderophore bacterial complexes (Dimkpa *et al*., 2009b). The role of endophytic siderophore-producing bacteria has been rarely studied; however, the ability to produce siderophores confers competitive advantages to endophytic bacteria for the colonization of plant tissues and the exclusion of other microorganisms from the same ecological niche (Loaces *et al*., 2011). These authors observed that the community of endophytic siderophore-producing bacteria associated to rice roots is richer than those from the soil at the tillering and grain-filling stages. Endophytic bacterial strains belonging to genus *Burkholderia* showed preferential localization inside rice plants, and their role may be relevant to prevent the infection of young plants by *Sclerotium oryzae* and *Rhizoctonia oryzae*.

In maize, endophytic strains belonging to genus *Bacillus* show different plant growth-promoting characteristics, such as siderophore production, and these effects were the most efficient against the growth of *Fusarium verticillioides, Colletotrichum graminicola, Bipolaris maydis*, and *Cercospora zea-maydis* fungi (Szilagyi-Zecchin *et al*., 2014). Siderophores produced by *A. brasilense* (REC2, REC3) showed *in vitro* antifungal activity against *Colletotrichum acutatum* (the causal agent of anthracnose). Also, a reduction of disease symptoms was observed in strawberry (*Fragaria vesca*) plants previously inoculated with *A. brasilense* (Tortora *et al*., 2011).

## ACC deaminase activity

Ethylene is an endogenously produced gaseous phytohormone that acts at low concentrations, participating in the regulation of all processes of plant growth, development and senescence (Shaharoona *et al*., 2006; Saleem *et al*., 2007). In addition to acting as a plant growth regulator, ethylene has also been identified as a stress phytohormone. Under abiotic and biotic stresses (including pathogen damage, flooding, drought, salt, and organic and inorganic contaminants), endogenous ethylene production is substantially accelerated and adversely affects the growth of the roots and thus the growth of the plant as a whole.

A number of mechanisms have been investigated aiming to reduce the levels of ethylene in plants. One of these mechanisms involves the activity of the bacterial enzyme 1-aminocyclopropane-1-carboxylate (ACC) deaminase (Glick, 2005; Jalili *et al*., 2009; Farajzadeh *et al*., 2012). ACC deaminase regulates the production of plant ethylene by metabolizing ACC (the immediate precursor of ethylene biosynthesis in higher plants) into α-ketobutyric acid and ammonia (Arshad *et al*., 2007; Saleem *et al*., 2007). A significant amount of plant ACC might be excreted from the plant roots and subsequently taken up by soil microorganisms and hydrolyzed by the enzyme ACC deaminase, thus decreasing the amount of ACC in the environment. When associated with plant roots, soil microbial communities with ACC deaminase activity might have a better growth than other free microorganisms, as these organisms use ACC as a source of nitrogen (Glick, 2005).

Bacterial ACC deaminase activity can be conceptually divided into two groups, based on high or low enzymatic activity (Glick, 2005). High ACC deaminase-expressing microorganisms nonspecifically bind to a variety of plant surfaces, and these microbes include rhizosphere and phyllosphere microorganisms and endophytes. However, low ACC deaminase-expressing microorganisms only bind to specific plants or are only present in certain tissues, and although these microbes do not lower the overall level of ethylene produced by the plant, they might prevent a localized increase in ethylene levels. Low ACC deaminase-expressing microorganisms include most, if not all, rhizobia species (Glick, 2005). Shaharoona *et al*. (2006) demonstrated that the co-inoculation of mung bean (*Vigna radiate* L.) with *Bradyrhizobium* and one bacterial strain presenting ACC deaminase activity enhanced nodulation as compared to inoculation with *Bradyrhizobium* alone, suggesting that this approach might be effective to achieve legume nodulation.

Onofre-Lemus *et al*. (2009) observed that ACC deaminase activity is a widespread feature in species belonging to genus *Burkholderia*. These authors identified 18 species of this genus exhibiting this activity; among these bacteria, *B. unamae* was able to endophytically colonize tomato (*Solanum lycopersicum* L.). In addition, tomato plants inoculated with the wild-type *B. unamae* strain presented better growth than those inoculated with a mutant strain deficient for ACC deaminase activity (Onofre-Lemus *et al*., 2009). In another study, a mutation in the ACC deaminase pathway altered the physiology of the endophytic *B. phytofirmans* PsJN2 strain, including the loss of ACC deaminase activity, an increase in IAA synthesis, a decrease in the production of siderophores and the loss of the ability to promote the growth of canola roots (*Brassica napus* L.; Sun *et al*., 2009).

Plant growth and productivity is negatively affected by abiotic stresses. Bal *et al*. (2013) demonstrated the effectiveness of bacteria exhibiting ACC deaminase activity, such as *Alcaligenes* sp., *Bacillus* sp., and *Ochrobactrum* sp., in inducing salt tolerance and consequently improving the growth of rice plants under salt stress conditions. Arshad *et al*. (2008) obtained similar results, demonstrating that a strain of *Pseudomonas* spp. with ACC deaminase activity partially eliminated the effect of drought stress on the growth of peas (*Pisum sativum* L.). Similarly, tomato plants pretreated with the endophytic bacteria *P. fluorescens* and *P. migulae* displaying ACC deaminase activity were healthier and showed better growth under high salinity stress compared with plants pretreated with an ACC deaminase-deficient mutant or without bacterial treatment (Ali *et al*., 2014). Moreover, the selection of endophytes with ACC deaminase activity could also be a useful approach for developing a successful phytoremediation strategy, given the potential of these bacteria to reduce plant stress (Glick, 2010).

## Phosphate solubilization

Phosphorus (P) is an essential nutrient for plants, participating as a structural component of nucleic acids, phospholipids and adenosine triphosphate (ATP), as a key element of metabolic and biochemical pathways, important particularly for BNF and photosynthesis (Khan *et al*., 2009; Richardson and Simpson, 2011). Plants absorb P in two soluble forms: the monobasic (H_2_PO_4_^−^) and the dibasic (HPO_4_^2−^) (Glass, 1989). However, a large proportion of P is present in insoluble forms and is consequently not available for plant nutrition. Low levels of P reflect the high reactivity of phosphate with other soluble components (Khan *et al*., 2009), such as aluminum in acid soils (pH < 5) and calcium in alkaline soils (pH > 7) (Holford, 1997; McLaughlin *et al*., 2011). Organic (incorporated into biomass or soil organic matter) and inorganic compounds, primarily in the form of insoluble mineral complexes, are major sources of available P in the soil (Rodríguez *et al*., 2006; Richardson and Simpson, 2011). Therefore, the availability of P depends on the solubility of this element, which could be influenced by the activity of plant roots and microorganisms in the soil. Phosphate-solubilizing bacteria and fungi constitute approximately 1-50% and 0.1-0.5%, respectively, of the total population of cultivable microorganisms in the soil (Chabot *et al*., 1993; Khan *et al*., 2009).

Among the different sources of P in the soil (as previously mentioned), the solubilization of inorganic phosphates has been the main focus of research studies. Phosphate-solubilizing bacteria solubilize inorganic soil phosphates, such as Ca_3_(PO_4_)_2_, FePO_4_, and AlPO_4_, through the production of organic acids, siderophores, and hydroxyl ions (Jones, 1998; Chen *et al*., 2006; Rodríguez *et al*., 2006, Sharma *et al*., 2013). Some bacteria only solubilize calcium phosphate, while other microorganisms are capable of solubilizing other forms of inorganic phosphates at different intensities. Bacterial isolates belonging to genera *Enterobacter, Pantoea* and *Klebsiella* solubilize Ca_3_(PO_4_)_2_ to a greater extent than FePO_4_ and AlPO_4_ (Chung *et al*., 2005). The production of organic acids, particularly gluconic and carboxylic, is one of the well-studied mechanisms utilized by microorganisms to solubilize inorganic phosphates (Rodriguez and Fraga, 1999).

Several phosphate-solubilizing bacteria have been isolated from the roots and rhizospheric soil of various plants (Ambrosini *et al*., 2012; Farina *et al*., 2012; Costa *et al*., 2013; Souza *et al*., 2013, 2014; Granada *et al*., 2013). Among the 336 strains associated with rice plants, Souza *et al*. (2013) identified 101 isolates belonging to the genera *Burkholderia, Cedecea, Cronobacter, Enterobacter, Pantoea* and *Pseudomonas* which were able to solubilize tricalcium phosphate [Ca_3_(PO_4_)_2_]. Ambrosini *et al*. (2012) demonstrated that *Burkholderia* strains associated with sunflower plants were predominant in Ca_3_(PO_4_)_2_ solubilization. Chen *et al*. (2006) had previously reported several phosphate-solubilizing bacteria strains belonging to the genera *Bacillus, Rhodococcus, Arthrobacter, Serratia, Chryseobacterium, Gordonia, Phyllobacterium* and *Delftia*. These authors also identified various types of organic acids produced by bacterial strains, such as the citric, gluconic, lactic, succinic and propionic acids.

Qin *et al*. (2011) suggested that the ability of rhizobia to solubilize inorganic phosphate is associated with rhizosphere acidification. Moreover, the inoculation with *Rhizobium* enhanced P acquisition by soybean plants, particularly where Ca_3_(PO_4_)_2_ was the primary P source. The inoculation of rice with the phosphate-solubilizing diazotrophic endophytes *Herbaspirillum* and *Burkholderia* increased grain yield and nutrient uptake in plants cultivated in soil with Ca_3_(PO_4_)_2_ and ^15^N-labeled fertilizer, suggesting that the selection and use of P-solubilizing diazotrophic bacteria is an effective strategy for the promotion of P solubilization (Estrada *et al*., 2013).

Several studies have reported the isolation of phosphate-solubilizing bacteria from soils or rhizospheres. Confirming that endophytes are important for phosphate solubilization, Chen *et al*. (2014) observed that the endophyte *Pantoea dispersa*, isolated from the roots of cassava (*Manihot esculenta* C.), effectively dissolved Ca_3_(PO_4_)_2_, FePO_4_, and AlPO_4_, producing salicylate, benzene-acetic and other organic acids. Moreover, the inoculation of *P. dispersa* in soil enhanced the concentration of soluble P in a microbial population, increasing the soil microbial diversity, which suggests that an endophyte could adapt to the soil environment and promote the release of P.

Soil also contains a wide range of organic substrates which can be a source of phosphorus for plant growth. Organic P forms, particularly phytates, are predominant in most soils (10-50% of total P) and mineralized by phytases (myo-inositol hexakisphosphate phosphohydrolases) (Rodríguez *et al*., 2006; Richardson and Simpson, 2011). Bacteria with phytase activity have been isolated from rhizosphere and proposed as PGPB to be used in soils with high content of organic P. Bacterial isolates identified as *Advenella* are positive for phytase production, and increased the P content and growth of Indian mustard (*Brassica juncea*) (Singh *et al*., 2014). In another study, Kumar *et al*. (2013) reported phytase-producing bacteria belonging to genera *Tetrathiobacter* and *Bacillus* which also promoted the growth of Indian mustard and significantly increased the P content. Idriss *et al*. (2002) reported that extracellular phytase from *B. amyloliquefaciens* FZB45 promoted the growth of maize seedlings. The production of phytase has been characterized in other rhizosphere bacteria, as for example, *Bacillus* sp., *Cellulosimicrobium* sp., *Acetobacter* sp., *Klebsiella terrigena, Pseudomonas* sp., *Paenibacillus* sp., and *Enterobacter* sp. (Yoon *et al*., 1996; Kerovuo *et al*., 1998; Idriss *et al*., 2002; Gulati *et al*., 2007; Acuña *et al*., 2011; Jorquera *et al*., 2011; Kumar *et al*., 2013; Singh *et al*., 2014). Moreover, bacteria with both activities, production of organic acids to solubilize inorganic P and production of phytase to mineralize phytate, have been isolated from the rhizospheres of different plants, such as perennial ryegrass (*Lolium perenne* L.), white clover (*Trifolium repens* L.), wheat, oat (*Avena sativa* L.) and yellow lupin (*Lupinus luteus* L.) (Jorquera *et al*., 2008).

## Inoculants Can Reduce Chemical Fertilization

The demand for chemical fertilizers in agriculture has historically been influenced by interrelated factors such as population growth worldwide, economic growth, agricultural production, among others (Morel *et al*., 2012). Interest in the use of inoculants containing PGPB that promote plant growth and yield has increased because nitrogen fertilizers are expensive and can damage the environment through water contamination with nitrates, acidification of soils and greenhouse-gas emissions (Adesemoye *et al*., 2009; Hungria *et al*., 2013). Plant-microorganism associations have long been studied, but their exploitation in agriculture for partially or fully replacing nitrogen fertilizers is still low (Hungria *et al*., 2013). Moreover, plants can only use a small amount of phosphate from chemical sources, because 75-90% of the added P is precipitated through metal-cation complexes and rapidly becomes fixed in soils (Sharma *et al*., 2013). Approximately 42 million tons of nitrogenous fertilizers are applied annually on a global scale for the production of the three major crop cereals: wheat, rice, and maize. Annually, 8 × 10^10^ kg of NH_3_ are produced by nitrogenous fertilizer industries, while 2.5 × 10^11^ kg of NH_3_ are fixed through BNF (Cheng, 2008). The nitrogen provided by BNF is less prone to leaching, volatilization and denitrification, as this chemical is used *in situ* and is therefore considered an important biological process that contributes to sustainable agriculture (Dixon and Kahn, 2004).

The management of bacteria, soil and plant interactions has emerged as a powerful tool in view of the biotechnological potential of these interactions, evidenced by increased crop productivity, reduction of production costs by reducing the volume of fertilizers applied and a better conservation of environmental resources. Moreover, inoculants are composed of beneficial bacteria that can help the plant meet its demands for nutrients. As previously discussed, these bacteria increase plant growth, accelerate seed germination, improve seedling emergence in response to external stress factors, protect plants from disease, and promote root growth using different strategies (Table 1). Whether gram-negative or gram-positive, these bacteria require isolation in culture media and analysis of various genotypic and phenotypic aspects, as well as analysis regarding their beneficial interaction with the host plant in experimental and natural conditions.

**Table 1:** Examples of PGPB used as inoculants or bacterial culture of different plant species in soil experiments.

Plant	Experimental conditions	Microorganism (s)	Main PGP-traits^3^	Main results	References
Chickpea^1^	Field: liquid culture applied to seed	*Pseudomonas jessenii* PS06, *Mesorhizobium ciceri* C-2/2	N_2_-fixing (C-2/2), P-solubilizing (PS06)	Plants inoculated with C-2/2, in single or dual inoculation, produced higher nodule fresh weight, nodule number and shoot N content, while PS06 had no significant effect on plant growth. However, the co-inoculation ranked highest in seed yield and nodule fresh weight.	Valverde *et al*. (2006)
	Pot and field: liquid culture applied to seed	*Rhizobium* spp., *B. subtilis* OSU-142, *Bacillus megaterium* M-3	N_2_-fixing (*Rhizobium*, OSU-142), biocontrol activity (OSU-142, M-3), P-solubilizing (M-3)	In the field, all the combined treatments containing *Rhizobium* were better for nodulation than the use of *Rhizobium* alone. Nodulation by native *Rhizobium* population was increased in single and dual inoculations with OSU-142 and M-3.	Elkoca *et al*. (2008)
Maize	Pot: liquid culture applied to seed or soil	*Burkholderia ambifaria* MCI 7	Siderophore, antifungal activity	The inoculation method influenced the plant growth: seed-applied liquid culture promoted increase on shoot fresh weight as the control, while soil-applied liquid culture reduced plant growth markedly.	Ciccillo *et al*. (2002)
	Pot: liquid culture applied to seed	*Pseudomonas alcaligenes* PsA15, *Bacillus polymyxa* BcP26, *Mycobacterium phlei* MbP18	N_2_-fixing, antifungal activity (BcP26 and MbP18), IAA (PsA15 and MbP18)	Bacterial inoculation had a much better stimulatory effect on plant growth and NPK content in nutrient-deficient soil than in nutrient-rich soil, where the bacterial inoculants stimulated only root growth and N, K uptake of the roots.	Egamberdiyeva (2007)
	Pot and field: liquid inoculant applied to seed	*A. brasilense* Ab-V5	N_2_-fixing, IAA	In pot trials with clay soil, plant growth was increased when Ab-V5 was applied at full dose. In sandy soil, nutrients and Ab-V5 were needed for a significant increase in the maize response. In the field, the grain production was increased when Ab-V5 and N were added, compared to N fertilization alone.	Ferreira *et al*. (2013)
Pea	Field: peat powder, granular, and liquid inoculant applied to seed or soil	*Rhizobium leguminosarum* bv. *viciae*	N_2_-fixing	The effects of inoculant formulation on nodule number, N accumulation and N_2_-fixation were: granular peat powder liquid = uninoculated. Soil-applied inoculant improved N nutrition of field pea compared to seed-applied inoculation, with or without applied urea-N.	Clayton *et al*. (2004)
	Pot: liquid culture applied to seed	*P. fluorescens* ACC-5 (biotype G) and ACC-14, *P. putida* Q-7 (biotype A)	ACC-deaminase	Rhizobacteria containing ACC-deaminase significantly decreased the “drought stress-imposed effects”, although with variable efficacy at different moisture levels. Strain ACC-5 greatly improved the water use efficiency at lowest soil moisture level.	Zahir *et al*. (2008)
Peanut^2^	Pot and field: liquid culture applied to seed	*P. fluorescens* PGPR1, PGPR2, and PGPR4	ACC-deaminase, IAA, siderophore, antifungal activity	Pod yield and NP contents in soil, shoot and kernel were significantly enhanced in treatments inoculated in pots, during rainy and post-rainy seasons. The PGPRs also significantly enhanced pod yield, haulm yield and nodule dry weight compared to controls, in 3 years of field trials.	Dey *et al*. (2004)
Rice	Field: peat inoculant applied to soil and seedling	*Pseudomonas* spp., *B. amyloliquefaciens, B. subtilis*,soil yeast	Not described	Inoculation significantly increased grain and straw yields and total N uptake, as well as grain quality in terms of N percentage. Inoculation was able to save 43 kg N ha^−1^, with an additional rice yield of 270 kg ha^−1^ in two consecutive rainy seasons at the experimental site.	Cong *et al*. (2009)
	Pot and field: liquid culture applied to seedling	*Azospirillum* sp. B510	N_2_-fixing, IAA	The field experiment indicated that inoculation with B510 increases tiller number, resulting in an increase in seed yield at commercial levels when compared with uninoculated plants.	Isawa *et al*. (2010)
	Field: liquid culture applied to seedling	*Azospirillum* sp. B510	N_2_-fixing, IAA	Growth in terms of tiller numbers and shoot length was significantly increased by inoculation. The application of *Azospirillum* sp. B510 not only enhanced rice growth, but also affected minor rice-associated bacteria.	Bao *et al*. (2013)
Soybean	Field: granular and peat inoculant applied to seed and in-furrow	*Bacillus cereus* UW85 (granular) and *B. japonicum* (peat)	Not described	The inoculation with UW85 resulted in stimulations in shoot dry weight, increased seed yield and seed N content, but the effect was site-specific. The stimulation in growth and N parameters by UW85 treatment was proportionally greater in the absence of *B. japonicum* inoculation than in the presence of the rhizobial inoculant.	Bullied *et al*. (2002)
	Field: liquid inoculant applied to seed and in-furrow	*B. japonicum* SEMIA 5079 and SEMIA 5080, *A. brasilense* Ab-V5 and Ab-V6	N_2_-fixing, IAA	Inoculation of seeds with rhizobia increased soybean yield by 8.4 %, and co-inoculation with *A. brasilense* in-furrow by an average 16.1 %. Seed co-inoculation with both microorganisms resulted in a mean yield increase of 14.1 % in soybean compared to the uninoculated control.	Hungria *et al*. (2013)
	Pot: liquid culture applied to seed	*B. amyloliquefaciens* sks_bnj_1	Siderophore, IAA, ACC-deaminase, antifungal activity, phytases	Inoculation significantly increased rhizosphere soil properties (enzyme activities, IAA production, microbial respiration, microbial biomass-C), and nutrient content in straw and seeds of soybean compared to uninoculated control.	Sharma *et al*. (2013)
Sugarcane	Pot and field: liquid culture applied to seedlings	*B. vietnamiensis* MG43, *G. diazotrophicus* LMG7603, *H. seropedicae* LMG6513	N_2_-fixing	Biomass increase due to MG43 inoculation reached 20% in the field. The inoculation of three strains was less effective than inoculation by a single MG43 suspension.	Govindarajan *et al*. (2006)
	Field: liquid culture applied to stem cuttings	*G. diazotrophicus* VI27	N_2_-fixing, siderophore, IAA, P-solubilizing	The strain showed a significant increase in the number of sets germinated, in the amount of soluble solids, and in the yield of sugarcane juice compared to control.	Beneduzi *et al*. (2013)
Wheat	Field: liquid inoculant applied to seed	*A. brasilense* INTA Az-39	N_2_-fixing, IAA	The inoculated crops exhibited more vigorous vegetative growth, with both greater shoot and root dry matter accumulation. Positive responses were found in about 70% of the experimental sites (total: 297 sites), independently of fertilization and other crop and soil management practices.	Díaz-Zorita and Fernández-Canigia (2009)
	Pot: liquid culture applied to soil	*B. subtilis* SU47, *Arthrobacter* sp. SU18	IAA, P-solubilizing	Sodium content was reduced under co-inoculation conditions but not after single inoculation with either strain or in the control. Plants grown under different salinity regimes and PGPR co-inoculation showed an increase in dry biomass, total soluble sugars and proline content, and reduced activity of antioxidant enzymes.	Upadhyay *et al*. (2012)
Wheat and maize	Field: peat and liquid inoculant applied to seed	*A. brasilense* Ab-V5 and Ab-V6	N_2_-fixing, IAA	Inoculants increased maize and wheat yields at low N fertilizer starter at sowing by 27% and 31%, respectively. A liquid inoculant containing a combination of the strains proved to be as effective as peat inoculant carrying the same strains, in both maize and wheat.	Hungria *et al*. (2010)

1 *Cicer arietinum* L.

2 *Arachis hypogaea* L.

3 When the characteristic is not displayed by all strains, those that present it are shown in parenthesis.

Rhizobia species are well investigated because of their symbiotic relationship with leguminous plants and their agronomical application as inoculants in the cultivation of economic crops (Alves *et al*., 2004; Torres *et al*., 2012). The soybean-*Bradyrhizobium* association is a good example of the efficiency of BNF, and *B*. *elkanii* and *B. japonicum* are species commonly used to inoculate this leguminous plant. In this system, the BNF is so efficient that attempts to increase grain yields by adding nitrogenous fertilizers are not successful in plants effectively inoculated with the recommended *Bradyrhizobium* strains (Alves *et al*., 2004). In Brazil, where approximately 70% of the nitrogenous fertilizers are imported, the costs of mineral N utilization in agriculture are high, and inoculants are a more cost-effective alternative, particularly for soybean crops. It is estimated that, in this culture alone, Brazil saves approximately US$ 7 billion per year thanks to the benefits of BNF (Hungria *et al*., 2013).

In the last few decades, a large array of bacteria associated with non-leguminous plants, including *Azospirillum* species, have demonstrated plant growth-promoting properties (Okon and Labandrera-Gonzalez, 1994; Garcia de Salamone *et al*., 1996; Bashan *et al*., 2004; Cassán and Garcia de Salamone, 2008; Hungria *et al*., 2010). *Azospirillum* might promote the growth, yield and nutrient uptake of different plant species of agronomic importance, particularly wheat and maize (Hungria *et al*., 2010). Inoculants containing *Azospirillum* have been tested under field conditions in Argentina, with positive results regarding plant growth and/or grain yield (Cassán and Garcia de Salamone, 2008). In Brazil, field experiments designed to evaluate the performance of *A. brasilense* strains isolated from maize plants showed effectiveness in both maize and wheat. These results were grounds for the authorization of the first strains of inoculants to be produced and commercially used in wheat and maize in this country. According to the authors (Hungria *et al*., 2010), the partial (50%) replacement of the nitrogenous fertilizer required for these crops in association with *Azospirillum* sp. inoculation would save an estimated US$ 1.2 billion per year, suggesting that the use of inoculants could reduce the use of chemical fertilizers worldwide.

Inoculation with a consortium of several bacterial strains could be an alternative to inoculation with individual strains, likely reflecting the different mechanisms used by each strain in the consortium. The co-inoculation of soybean and common bean (*P. vulgaris* L.) with rhizobia and *A. brasilense* inoculants showed good results for improving sustainability (Hungria *et al*., 2013). In field trials, the co-inoculation of soybean with *B. japonicum* and *A. brasilense* species resulted in outstanding increases in grain yield and improved nodulation compared with the non-inoculated control. For common bean, co-inoculation with *Rhizobium tropici* and *A. brasilense* species resulted in an impressive increase in grain yield, varying from 8.3% when *R. tropici* was inoculated alone to 19.6% when the two bacterial species were used. Domenech *et al*. (2006) showed that the inoculation of tomato and pepper with a product based on *Bacillus subtilis* GB03 (a growth-promoting agent), *B*. *amyloliquefaciens* IN937a (endophytic bacteria, systemic resistance inducer) and chitosan, combined with different bacterial strains such as *P. fluorescens*, provided better biocontrol against *Fusarium* wilt and *Rhizoctonia* damping-off as compared to the use of the product alone.

## Processes Involved in the Efficiency of Inoculation

### Exudation by plant roots

Plant roots respond to environmental conditions through the secretion of a wide range of compounds, according to nutritional status and soil conditions (Cai *et al*., 2012; Carvalhais *et al*., 2013). This action interferes with the plant-bacteria interaction and is an important factor contributing to the efficiency of the inoculant (Cai *et al*., 2009, 2012; Carvalhais *et al*., 2013). Root exudation includes the secretion of ions, free oxygen and water, enzymes, mucilage, and a diverse array of C-containing primary and secondary metabolites (Bais *et al*., 2006). The roots of plants excrete 10-44% of photosynthetically fixed C, which serves as energy source, signaling molecules or antimicrobials for soil microorganisms (Guttman *et al*., 2014). The root exudation varies with plant age and genotype, and consequently specific microorganisms respond and interact with different host plants (Bergsma-Vlami *et al*., 2005; Aira *et al*., 2010; Ramachandran *et al*., 2011). Thus, inoculants are generally destined to the one specific plant from which the bacterium was isolated.

The well-studied flavonoids also vary with plant age and physiological state when exuded from legume rhizospheres, and induce *nodD* gene expression in rhizobial strains. NodD is a transcriptional activator of bacterial genes involved in the infection and nodule formation during the establishment of legume-rhizobia symbioses (Long, 2001). Similarly to flavonoids, several compounds secreted by roots modulate the relationships between plants and PGPB (Badri *et al*., 2009). *Bacillus subtilis* FB17, for instance, is attracted by L-malic acid, secreted by the roots of *Arabidopsis thaliana* infected with the foliar pathogen *P. syringae* pv *tomato* (Pst DC3000; Rudrappa *et al*., 2008). Profiles of secreted secondary metabolites, such as phenolic compounds, flavonoids and hydroxycinnamic derivatives, were different in rice cultivars (Nipponbare and Cigalon), according to inoculation with *Azospirillum* 4B and B510 strains. Interestingly, strains 4B and B510 preferentially increased the growth of the cultivar from which they were isolated; however, both strains effectively colonized either at the rhizoplane (4B and B510) or inside roots (B510) (Chamam *et al*., 2013).

Some molecules exudated from roots might act as antimicrobial agents against one organism and as stimuli for the establishment of beneficial interactions with regard to other organisms. For example, canavanine, a non-protein amino acid analog to arginine, secreted at high concentrations by many varieties of legume seeds, acts as an antimetabolite in many biological systems and also stimulates the adherence of rhizobia that detoxify this compound (Cai *et al*., 2009). In the Leguminosae family, canavanine is a major N storage compound in the seeds of many plants, and constitutes up to 13% of the dry weight of seeds (Rosenthal, 1972). Benzoxazinoids (BXs) are secondary metabolites synthesized by Poaceae during early plant growth stages. These molecules are effective in plant defense and allelopathy (Nicol *et al*., 1992; Zhang *et al*., 2000; Frey *et al*., 2009; Neal *et al*., 2012). However, qualitative and quantitative modifications of BXs production in maize were differentially induced according to inoculation with different *Azospirillum* strains (Walker *et al*., 2011a, b).

The quantitative and qualitative changes in the composition of the exudates result from the activation of biochemical defense systems through elicitors mimicking stresses in plants. Biotic and abiotic elicitors stimulate defense mechanisms in plant cells and greatly increase the diversity and amount of exudates (Cai *et al*., 2012). Several studies have reported that endophytic PGPB induce stress and defense responses, causing changes in plant metabolites that lead to the fine control of bacterial populations inside plant tissues (Miché *et al*., 2006; Chamam *et al*., 2013; Straub *et al*., 2013). Jasmonate is an important plant-signaling molecule that mediates biotic and abiotic stress responses and aspects of growth and development (Wasternack, 2007). Rocha (2007) showed that the jasmonate response is initiated prior to the establishment of an effective association. Following the inoculation of *Miscanthus sinensis* with *H. frisingense* GSF30T, transcriptome and proteome data showed the rapid and strong up-regulation of jasmonate-related genes in plants, and this effect was suppressed after the establishment of an association with bacteria (Straub *et al*., 2013).

### Bacterial root colonization

Rhizosphere competence reflects variation in the ability of a PGPB to colonize plant roots during the transition from free-living to root-associated lifestyles. The attachment and colonization of roots are modulated through PGPB abilities involved in important processes for survival, growth, and function in soil (Cornforth and Foster, 2013). Associative and endophytic PGPB respond to plant exudates through the modulation of the expression of several genes, such as those associated with exopolysaccharide (EPS) biosynthesis and biofilm formation (Rudrappa *et al*., 2008; Meneses *et al*., 2011; Beauregard *et al*., 2013). Biofilms are surface-adherent microbial populations typically embedded within a self-produced matrix material (Fuqua and Greenberg, 2002). As previously mentioned, *B. subtilis* is attracted by L-malic acid secreted by *A. thaliana*. Moreover, bacterial biofilm formation is selectively triggered through L-malic acid, in a process dependent on the same gene matrix required for *in vitro* biofilm formation (Rudrappa *et al*., 2008; Beauregard *et al*., 2013). EPS biosynthesis is also required for biofilm formation and plant colonization by the endophyte *G. diazotrophicus*. A functional mutant of *G. diazotrophicus* PAL5 for EPS production did not attach to the rice root surface or exhibit endophytic colonization (Meneses *et al*., 2011).

Cell-cell communication via quorum sensing (QS) regulates root colonization and biocontrol (Danhorn and Fuqua, 2007). Quorum sensing involves intercellular signaling mechanisms that coordinate bacterial behavior, host colonization and stress survival to monitor population density (Danhorn and Fuqua, 2007; Schenk *et al*., 2012; He and Bauer, 2014). Plant-associated bacteria frequently employ this signaling mechanism to modulate and coordinate interactions with plants, including acylated homoserine lactones (AHLs) among proteobacteria and oligopeptides among gram-positive microbes (Danhorn and Fuqua, 2007). The endophytic *G. diazotrophicus* PAL5 strain colonizes a broad range of host plants, presenting QS comprising *luxR* and *luxI* homolog gene products and producing eight molecules of the AHL family (Bertini *et al*., 2014). The levels of QS were modified according to glucose concentration, the presence of other C sources and saline stress conditions.

Stress-induced bacterial genes are also associated with plant-bacterial interactions. The bacterial enzymes superoxide dismutase and glutathione reductase were crucial for the endophytic colonization of rice roots by *G. diazotrophicus* PAL5 (Alquéres *et al*., 2013). *Bacillus amyloliquefaciens* FZB42 genes involved in chemotaxis and motility were induced through exudates from P-deficient maize plants, whereas the exudates from N-deficient plants triggered a general bacterial stress response (Carvalhais *et al*., 2013). The global gene expression of *A. lipoferum* 4B cells during interactions with different rice cultivars (Nipponbare and Cigalon) involved genes associated with reactive oxygen species (ROS) detoxification, multidrug efflux, and complex regulatory networks (Drogue *et al*., 2014). The cultivar-specific expression profiles of PGPB suggested host-specific adaptation (Drogue *et al*., 2014).

Moreover, microbial competition was closely associated with the root colonization efficiency of PGPB, as the exudation of different compounds attracts a great number of different microbial populations. The deposition of nutrients in the plant rhizosphere (rhizodeposition) supports higher microbial growth than the surrounding soil, a phenomenon referred to as the “rhizosphere effect” (Rovira, 1965; Dunfield and Germida, 2003; Mougel *et al*., 2006). This intense molecular communication surrounding the roots provides a broad range of microbe-microbe interactions, making this environment highly competitive among soil bacteria. Microbial competition and activities include, for example, motility (Capdevila *et al*., 2004; de Weert *et al*., 2002), attachment (Buell and Anderson, 1992; Rodriguez-Navarro *et al*., 2007), growth (Browne *et al*., 2009; Miller *et al*., 2010), stress resistance (Espinosa-Urgel *et al*., 2000; Martinez *et al*., 2009), secondary metabolite production (Abbas *et al*., 2002; Haas and Defago, 2005), and quorum sensing (Edwards *et al*., 2009; Ramachandran *et al*., 2011).

### Soil health

Soil is a heterogeneous mixture of different organisms and organic and mineral substances present in three phases: solid, liquid, and gaseous (Kabata-Pendias, 2004). The physical forces and natural grouping of particles result in the formation of soil aggregates of different sizes, arrangements and stabilities, which are the basic units of soil structure (Lynch and Bragg, 1985). Soil aggregation is influenced by several factors, such as soil mineralogy, cycles of wetting and drying, the presence of iron and aluminum oxides as a function of soil pH range, and clay and organic material contents (Lynch and Bragg, 1985; Cammeraat and Imeson, 1998; Castro Filho *et al*., 2002; Majumder and Kuzyakov, 2010; Vogel *et al*., 2014). Plant roots directly contribute to the stability of soil aggregates through the inherent abundance of these structures in organic matter and the production of exudates stimulating microbial activity, and indirectly by the production of EPS (Leigh and Coplin, 1992; Alami *et al*., 2000; Schmidt *et al*., 2011).

The fine spatial heterogeneity of soils results in a complex mosaic of gradients selecting for or against bacterial growth (Vos *et al*., 2013). The microbial biomass decreases with soil depth, and changes in the community composition reflect substrate specialization (Schmidt *et al*., 2011). The distribution of micro (< 250 μm) and macro (> 250 μm)-aggregates provides microhabitats differentially assembled in terms of temperature, aeration, water retention and movement (Dinel *et al*., 1992; Zhang, 1994; Denef *et al*., 2004; Schmidt *et al*., 2011). Soil aggregates of different pore sizes influence C sequestration and the availability of nutrients (Zhang *et al*., 2013), and low pore connectivity due to low water potential increases the diversity of bacterial communities in the soil (Carson *et al*., 2010; Ruamps *et al*., 2011). Moreover, the moisture content, pore size and habitat connectivity differently impact the expansion of motile rod-shaped and filamentous bacterium types (Wolf *et al*., 2013).

Soil stability results from a combination of biotic and abiotic characteristics, and the microbial communities could provide a quantitative measure of soil health, as these bacteria determine ecosystem functioning according to biogeochemical processes (Griffiths and Philippot, 2012). Soil health defines the capacity of soil to function as a vital living system, within ecosystem and land-use boundaries, to sustain plant and animal productivity, maintain or enhance water and air quality, and promote plant and animal health (Doran and Zeiss, 2000). The factors controlling broad-range soil health comprise chemical, physical, and biological features, such as soil type, climate, cropping patterns, use of pesticides and fertilizers, availability of C substrates and nutrients, toxic material concentrations, and the presence or absence of specific assemblages and types of organisms (Doran and Zeiss, 2000; Young and Ritz, 1999; Kibblewhite *et al*., 2008).

The sustainable management of soils requires soil monitoring, including biological indicators such as microbial communities, which provide many potentially interesting indicators for environmental monitoring in response to a range of stresses or disturbances (Pulleman *et al*., 2012). Community stability is a functional property that focuses on community dynamics in response to perturbation: the return to a state of equilibrium following perturbation is the ability to resist to changes, which is called resistance; the rate of return to a state of equilibrium following perturbation is called resilience (Robinson *et al*., 2010). Soil functional resilience is governed by the effects of the physicochemical structure on microbial community composition and physiology (Griffiths *et al*., 2008). Microbial catabolic diversity is reduced through intensive land-use, which may have implications for the resistance of the soils to stress or disturbance (Degens *et al*., 2001; Ding *et al*., 2013).

Modern land-use practices highly influence the factors controlling soil health because, while these techniques increase the short-term supply of material goods, over time these practices might undermine many ecosystem services on regional and global scales (Foley *et al*., 2005). Soil disturbances operate at various spatial and temporal scales and mediate soil spatial heterogeneity. For instance, such disturbances may reduce the biomass of dominant organisms or provoke alterations in the physical structure of the soil substrate (Ettema and Wardle, 2002). Cultivation intensity reduces C content and changes the distribution and stability of soil aggregates, leading to a loss of C-rich macroaggregates and an increase of C-depleted microaggregates in soils (Six *et al*., 2000). Moreover, soil aggregation is a major ecosystem process directly impacted, via intensified land-use, by soil disturbances, or indirectly through impacts on biotic and abiotic factors that affect soil aggregates (Barto *et al*., 2010).

The physical disruption spectrum induced by tillage presents various degrees of soil disturbance and associations between no-tillage or minimum tillage and the ‘beneficial’ effects on soil microorganisms (Elliott and Coleman, 1988; Derpsch *et al*., 2014). The primary effect of tillage is the physical disturbance of the soil profile through alterations in the habitat space, water and substrate distribution and the spatial arrangement of pore pathways (Young and Ritz, 1999). Accumulated evidence suggests that conserved tillage systems, including no-tillage and reduced tillage, effectively reverse the disadvantage of conventional tillage in depleting the carbon stock through increases in the abundance and activity of the soil biota (Zhang *et al*., 2013). Lower microbial biomass in arable land likely reflects soil disturbance through tillage and the tillage-induced changes in soil properties (Cookson *et al*., 2008).

Disturbances alter the immediate environment, potentially leading to repercussions or direct alterations to this community (Shade *et al*., 2012). The manipulation of soil structure is one of the principal mechanisms for the regulation of microbial dynamics, at both the small and field scale (Elliott and Coleman, 1988; Derpsch *et al*., 2014). The microscale impact in crop soil under grassland, tillage, and no-tillage systems resulted in micro-aggregates containing similar bacterial communities, despite the land management practice, whereas strong differences were observed between communities inhabiting macro-aggregates (Constancias *et al*., 2014). In this same study, tillage decreased the density and diversity of bacteria from 74 to 22% and from 11 to 4%, respectively, and changed taxonomic groups in micro and macro-aggregates. These changes led to the homogenization of bacterial communities, reflecting the increased protection of micro-aggregates.

The combination of crop rotation with legumes, tillage management and soil amendments considerably influences the microbiotic properties of soil. Conventional agriculture systems, according to the FAO definition, use no tillage and have seeds placed at a proper depth in untilled soil, with previous crops or cover crop residues retained on the soil surface. In a no-tillage system, crop residue management plays an equally important role in minimizing and even avoiding soil disturbance (Derpsch *et al*., 2014). More research will expand our understanding of the combined effects of these alternatives on feedback between soil microbiotic properties and soil organic C accrual (Ghimire *et al*., 2014). However, the terms “reduced tillage” or “minimum tillage” or other degrees of tillage disturbance have been coined as no-tillage systems, and this term has been revised to “conservation agriculture systems” as a more holistic description (Derpsch *et al*., 2014). These authors also revised other terms associated with no-tillage systems.

## Conclusions

At a global scale, the effects of continuous agricultural practices such as fertilization can cause serious damage to the environment. Inoculation is one of the most important sustainable practices in agriculture, because microorganisms establish associations with plants and promote plant growth by means of several beneficial characteristics. Endophytes are suitable for inoculation, reflecting the ability of these organisms for plant colonization, and several studies have demonstrated the specific and intrinsic communication among bacteria and plant hosts of different species and genotypes.

The combination of different methodologies with these bacteria, such as identification of plant growth-promoting characteristics, the identification of bacterial strains, as well as assays of seed inoculation in laboratory conditions and cultivation experiments in the field, are part of the search for new technologies for agricultural crops. Thus, when this search shows a potential bacterial inoculant, adequate for reintroduction in the environment, many genera such as *Azospirillum, Bacillus* and *Rhizobium* may be primary candidates.

Finally, the search for beneficial bacteria is important for the development of new and efficient inoculants for agriculture. Also important are investments in technologies that can contribute to increase the inoculum efficiency and the survival rate of bacteria adherent to the seeds, which are other essential factors for successful inoculation. Thus, the introduction of beneficial bacteria in the soil tends to be less aggressive and cause less impact to the environment than chemical fertilization, which makes it a sustainable agronomic practice and a way of reducing the production costs.

## References

[B1] Abbas A, Morrissey JP, Marquez PC, Sheehan MM, Delany IR, O'Gara F (2002). Characterization of interactions between the transcriptional repressor
PhlF and its binding site at the *phlA* promoter in
*Pseudomonas fluorescens* F113. J Bacteriol.

[B2] Acuña JJ, Jorquera MA, Martínez OA, Menezes-Blackburn D, Fernández MT, Marschner P, Greiner R, Mora ML (2011). Indole acetic acid and phytase activity produced by rhizosphere
bacilli as affected by pH and metals. J Soil Sci Plant Nutr.

[B3] Adesemoye AO, Torbert HA, Kloepper JW (2009). Plant growth-promoting rhizobacteria allow reduced application rates
of chemical fertilizers. Microb Ecol.

[B4] Aira M, Gómez-Brandón M, Lazcano C, Baath E, Domínguez J (2010). Plant genotype strongly modifies the structure and growth of maize
rhizosphere microbial communities. Soil Biol Biochem.

[B5] Akbari GA, Arab SM, Alikhani HA, Allahdadi I, Arzanesh MH (2007). Isolation and selection of indigenous *Azospirillum*
spp. and the IAA of superior strains effects on wheat roots. World J Agric Sc.

[B6] Alami Y, Achouak W, Marol C, Heulin T (2000). Rhizosphere soil aggregation and plant growth promotion of sunflowers
by an exopolysaccharide-producing *Rhizobium* sp. strain isolated
from sunflower roots. Appl Environ Microbiol.

[B7] Ali S, Trevor CC, Glick BR (2014). Amelioration of high salinity stress damage by plant growth-promoting
bacterial endophytes that contain ACC deaminase. Plant Physiol Biochem.

[B8] Alquéres S, Meneses C, Rouws L, Rothballer M, Baldani I, Schmid M, Hartmann A (2013). The bacterial superoxide dismutase and glutathione reductase are
crucial for endophytic colonization of rice roots by *Gluconacetobacter
diazotrophicus* PAL5. Mol Plant-Microbe Interact.

[B9] Alves BJR, Boddey RM, Urquiaga S (2004). The success of BNF in soybean in Brazil. Plant Soil.

[B10] Ambrosini A, Beneduzi A, Stefanski T, Pinheiro FG, Vargas LK, Passaglia LMP (2012). Screening of plant growth promoting rhizobacteria isolated from
sunflower (*Helianthus annuus* L.). Plant Soil.

[B11] Andrews SC, Robinson AK, Rodríguez-Quinõnes F (2003). Bacterial iron homeostasis. FEMS Microbiol Rev.

[B12] Arnold W, Rump A, Klipp W, Priefer UB, Pühler A (1988). Nucleotide sequence of a 24,206-base-pair DNA fragment carrying the
entire nitrogen fixation gene cluster of *Klebsiella pneumoniae*. J Mol Biol.

[B13] Arshad M, Saleem M, Hussain S (2007). Perspectives of bacterial ACC deaminase in
phytoremediation. Trends Biotechnol.

[B14] Arshad M, Shaharoona B, Mahmood T (2008). Inoculation with *Pseudomonas* spp. containing
ACC-deaminase partially eliminates the effects of drought stress on growth, yield,
and ripening of pea (*Pisum sativum* L.). Pedosphere.

[B15] Bacon CW, Hinton DM, Gnanamanickam SS (2006). Bacterial endophytes: The endophytic niche, its
occupants, and its utility. Plant-Associated Bacteria.

[B16] Badri DV, Vivanco JM (2009). Regulation and function of root exudates. Plant Cell Environ.

[B17] Bais HP, Weir TL, Perry LG, Gilroy S, Vivanco JM (2006). The role of root exudates in rhizosphere interactions with plants and
other organisms. Annu Rev Plant Biol.

[B18] Baldani JI, Caruso L, Baldani VLD, Goi SR, Döbereiner J (1997). Recent advances in BNF with non-legume plants. Soil Biol Biochem.

[B19] Bal HB, Nayak L, Das S, Adhya TK (2013). Isolation of ACC deaminase producing PGPR from rice rhizosphere and
evaluating their plant growth promoting activity under salt stress. Plant Soil.

[B20] Bao Z, Sasaki K, Okubo T, Ikeda S, Anda M, Hanzawa E, Kaori K, Tadashi S, Hisayuki M, Minamisawa K (2013). Impact of *Azospirillum* sp. B510 inoculation on
rice-associated bacterial communities in a paddy field. Microbes Environ.

[B21] Barto EK, Alt F, Oelmann Y, Wilcke W, Rillig MC (2010). Contributions of biotic and abiotic factors to soil aggregation across
a land use gradient. Soil Biol Biochem.

[B22] Bashan Y, Holguin G, De-Bashan LE (2004). *Azospirillum*-plant relations physiological, molecular,
agricultural, and environmental advances (1997-2003). Can J Microbiol.

[B23] Baudoin E, Lerner A, Mirza MS, Zemrany HE, Prigent-Combaret C, Jurkevich E, Spaepen S, Vanderleyden J, Nazaret S, Okon Y (2010). Effects of *Azospirillum brasilense* with genetically
modified auxin biosynthesis gene *ipdC* upon the diversity of the
indigenous microbiota of the wheat rhizosphere. Res Microbiol.

[B24] Beauregard PB, Chai Y, Vlamakis H, Losick R, Kolter R (2013). *Bacillus subtilis* biofilm induction by plant
polysaccharides. Proc Natl Acad Sci USA.

[B25] Beneduzi A, Moreira F, Costa PB, Vargas LK, Lisboa BB, Favreto R, Baldani JI, Passaglia LMP (2013). Diversity and plant growth promoting evaluation abilities of bacteria
isolated from sugarcane cultivated in the South of Brazil. Appl Soil Ecol.

[B26] Bergsma-Vlami M, Prins ME, Raaijmakers JM (2005). Influence of plant species on population dynamics, genotypic diversity
and antibiotic production in the rhizosphere by indigenous
*Pseudomonas* spp. FEMS Microbiol Ecol.

[B27] Bertalan M, Albano R, de Pádua V, Rouws L, Rojas C, Hemerly A, Teixeira K, Schwab S, Araujo J, Oliveira A (2009). Complete genome sequence of the sugarcane nitrogen-fixing endophyte
*Gluconacetobacter diazotrophicus* PAl5. BMC Genomics.

[B28] Bertini EV, Peñalver CGN, Leguina AC, Irazusta VP, De Figueroa LI (2014). *Gluconacetobacter diazotrophicus* PAL5 possesses an active quorum
sensing regulatory system. Antonie van Leeuwenhoek.

[B29] Boucher Y, Douady CJ, Papke RT, Walsh DA, Boudreau MER, Nesbo CL, Case J, Doolittle WF (2003). Lateral gene transfer and the origins of prokaryotic
groups. Annu Rev Genet.

[B30] Bullied JW, Buss WTJ, Vessey KJ (2002). *Bacillus cereus* UW85 inoculation effects on growth, nodulation,
and N accumulation in grain legumes: Field studies. Can J Plant Sci.

[B31] Browne P, Rice O, Miller SH, Burke J, Dowling DN, Morrissey JP, O'Gara F (2009). Superior inorganic phosphate solubilization is linked to phylogeny
within the *Pseudomonas fluorescens* complex. Appl Soil Ecol.

[B32] Buell CR, Anderson AJ (1992). Genetic analysis of the *aggA* locus involved in
agglutination and adherence of *Pseudomonas putida*, a beneficial
fluorescent pseudomonad. Mol Plant Microbe Interact.

[B33] Cai T, Cai W, Zhang J, Zheng H, Tsou AM, Xiao L, Zhong Z, Zhu J (2009). Host legume-exuded antimetabolites optimize the symbiotic
rhizosphere. Mol Microbiol.

[B34] Cai Z, Kastell A, Knorr D, Smetanska I (2012). Exudation: An expanding technique for continuous production and
release of secondary metabolites from plant cell suspension and hairy root
cultures. Plant cell reports.

[B35] Cammeraat LH, Imeson AC (1998). Deriving indicators of soil degradation from soil aggregation studies
in southeastern Spain and southern France. Geomorphology.

[B36] Capdevila S, Martinez-Granero FM, Sanchez-Contreras M, Rivilla R, Martin M (2004). Analysis of *Pseudomonas fluorescens* F113 genes
implicated in flagellar filament synthesis and their role in competitive root
colonization. Microbiology-SGM.

[B37] Carson JK, Gonzalez-Quiñones V, Murphy DV, Hinz C, Shaw JA, Gleeson DB (2010). Low pore connectivity increases bacterial diversity in
soil. Appl Environ Microbiol.

[B38] Carvalhais LC, Dennis PG, Fan B, Fedoseyenko D, Kierul K, Becker A, Von Wiren N, Borriss R (2013). Linking plant nutritional status to plant-microbe
interactions. PLoS One.

[B39] Cassán FD, Garcia de Salamone I (2008). *Azospirillum* sp.: Cell Physiology, Plant Interactions and
Agronomic Research in Argentina.

[B40] Castro C, Lourenço A, De F Guimarães M, Fonseca ICB (2002). Aggregate stability under different soil management systems in a red
latosol in the state of Parana, Brazil. Soil Tillage Res.

[B41] Chabot R, Antoun H, Cescas MP (1993). Stimulation de la croissance du mais et de la laitue romaine par
desmicroorganismes dissolvant le phosphore inorganique. Can J Microbiol.

[B42] Chamam A, Sanguin H, Bellvert F, Meiffren G, Comte G, Wisniewski-Dyé F, Bertrand C, Prigent-Combaret C (2013). Plant secondary metabolite profiling evidences strain-dependent effect
in the *Azospirillum-Oryza sativa* association. Phytochemistry.

[B43] Cheng Q (2008). Perspectives in biological nitrogen fixation research. J Integr Plant Biol.

[B44] Chen YP, Rekha PD, Arun AB, Shen FT, Lai WA, Young CC (2006). Phosphate solubilizing bacteria from subtropical soil and their
tricalcium phosphate solubilizing abilities. Appl Soil Ecol.

[B45] Chen Y, Fan JB, Du L, Xu H, Zhang QY, He YQ (2014). The application of phosphate solubilizing endophyte *Pantoea
dispersa* triggers the microbial community in red acidic
soil. Appl Soil Ecol.

[B46] Chung H, Park M, Madhaiyana M, Seshadri S, Song J, Cho H, Sa T (2005). Isolation and characterization of phosphate solubilizing bacteria from
the rhizosphere of crop plants of Korea. Soil Biol Biochem.

[B47] Ciccillo F, Fiore A, Bevivino A, Dalmastri C, Tabacchioni S, Chiarini L (2002). Effects of two different application methods of *Burkholderia
ambifaria* MCI7 on plant growth and rhizospheric bacterial
diversity. Environ Microbiol.

[B48] Clayton GW, Rice WA, Lupwayi NZ, Johnston AM, Lafond GP, Grant CA, Walley F (2004). Inoculant formulation and fertilizer nitrogen effects on field pea:
Nodulation, N_2_ fixation and nitrogen partitioning. Can J Plant Sci.

[B49] Cong PT, Dung TD, Hien TM, Hien NT, Choudhury AT, Kecskés ML, Kennedy IR (2009). Inoculant plant growth-promoting microorganisms enhance utilisation of
urea-N and grain yield of paddy rice in southern Vietnam. Eur J Soil Biol.

[B50] Constancias F, Prévost-Bouré NC, Terrat S, Aussems S, Nowak V, Guillemin JP, Bonnotte A, Biju-Duval L, Navel A, Martins JMF (2014). Microscale evidence for a high decrease of soil bacterial density and
diversity by cropping. Agron Sustainable Dev.

[B51] Cookson WR, Murphy DV, Roper MM (2008). Characterizing the relationships between soil organic matter
components and microbial function and composition along a tillage disturbance
gradient. Soil Biol Biochem.

[B52] Cornforth DM, Foster KR (2013). Competition sensing: The social side of bacterial stress
responses. Nat Rev Microbiol.

[B53] Costa P, Beneduzi A, Souza R, Schoenfeld R, Vargas LK, Passaglia LMP (2013). The effects of different fertilization conditions on bacterial plant
growth promoting traits: Guidelines for directed bacterial prospection and
testing. Plant Soil.

[B54] Costa PB, Granada CE, Ambrosini A, Moreira F, Souza R, Passos JFM, Arruda L, Passaglia LMP (2014). A model to explain plant growth promotion traits: A multivariate
analysis of 2,211 bacterial isolates. PLoS One.

[B55] Danhorn T, Fuqua C (2007). Biofilm formation by plant-associated bacteria. Annu Rev Microbiol.

[B56] Denef K, Six J, Merckx R, Paustian K (2004). Carbon sequestration in microaggregates of no-tillage soils with
different clay mineralogy. Soil Sci Soc Am J.

[B57] Degens BP, Schipper LA, Sparling GP, Duncan LC (2001). Is the microbial community in a soil with reduced catabolic diversity
less resistant to stress or disturbance?. Soil Biol Biochem.

[B58] Derpsch R, Franzluebbers AJ, Duiker SW, Reicosky DC, Koeller K, Friedrich T, Sturny WG, Sá JCM, Weiss K (2014). Why do we need to standardize no-tillage research?. Soil Tillage Res.

[B59] Dey R, Pal KK, Bhatt DM, Chauhan SM (2004). Growth promotion and yield enhancement of peanut (*Arachis
hypogaea* L.) by application of plant growth-promoting
rhizobacteria. Microbiol Res.

[B60] de Weert S, Vermeiren H, Mulders IHM, Kuiper I, Hendrickx N, Bloemberg GV, Vanderleyden J, De Mot R, Lugtenberg BJJ (2002). Flagella-driven chemotaxis towards exudate components is an important
trait for tomato root colonization by *Pseudomonas fluorescens*. Mol Plant Microbe Interact.

[B61] Díaz-Zorita M, Fernández-Canigia MV (2009). Field performance of a liquid formulation of *Azospirillum
brasilense* on dryland wheat productivity. Eur J Soil Biol.

[B62] Dimkpa C, Weinand T, Asch F (2009a). Plant-rhizobacteria interactions alleviate abiotic stress
conditions. Plant Cell Environ.

[B63] Dimkpa CO, Merten D, Svatos A, Büchel G, Kothe E (2009b). Siderophores mediate reduced and increased uptake of cadmium by
*Streptomyces tendae* F4 and sunflower (*Helianthus
annuus*), respectively. J Appl Microbiol.

[B64] Dinel H, Levesque PEM, Jambu P, Righi D (1992). Microbial activity and long-chain aliphatics in the formation of
stable soil aggregates. Soil Sci Soc Am J.

[B65] Ding GC, Piceno YM, Heuer H, Weinert N, Dohrmann AB, Carrillo A, Andersen GL, Castellanos T, Tebbe CC, Smalla K (2013). Changes of soil bacterial diversity as a consequence of agricultural
land use in a semi-arid ecosystem. PLoS One.

[B66] Dixon R, Kahn D (2004). Genetic regulation of biological nitrogen fixation. Nat Rev Microbiol.

[B67] Dobbelaere S, Croonenborghs A, Thys A, Broek AV, Vanderleyden J (1999). Phytostimulatory effect of *Azospirillum brasilense*
wild type and mutante strains altered in IAA production on wheat. Plant Soil.

[B68] Domenech J, Reddy MS, Kloepper JW, Ramos B, Gutierrez-Mañero J (2006). Combined application of the biological product LS213 with
*Bacillus, Pseudomonas* or *Chryseobacterium* for
growth promotion and biological control of soil-borne diseases in pepper and
tomato. BioControl.

[B69] Doran JW, Zeiss MR (2000). Soil health and sustainability: Managing the biotic component of soil
quality. Appl Soil Ecol.

[B70] Drogue B, Sanguin H, Borland S, Prigent-Combaret C, Wisniewski-Dyé F (2014). Genome wide profiling of *Azospirillum lipoferum* 4B
gene expression during interaction with rice roots. FEMS Microbiol Ecol.

[B71] Dunfield KE, Germida JJ (2003). Seasonal changes in the rhizosphere microbial communities associated
with field-grown genetically modified canola (*Brassica
napus*). Appl Environ Microbiol.

[B72] Edwards A, Frederix M, Wisniewski-Dye F, Jones J, Zorreguieta A, Downie JA (2009). The cin and rai quorum-sensing regulatory systems in *Rhizobium
leguminosarum* are coordinated by ExpR and CinS, a small regulatory
protein coexpressed with CinI. J Bacteriol.

[B73] Egamberdiyeva D (2007). The effect of plant growth promoting bacteria on growth and nutrient
uptake of maize in two different soils. Appl Soil Ecol.

[B74] Elbeltagy A, Nishioka K, Sato T, Suzuki H, Ye B, Hamada T, Isawa T, Mitsui H, Minamisawa K (2001). Endophytic colonization and in plant nitrogen fixation by a
sHerbaspirillum sp isolated from wild rice species.. Appl Environ Microbiol.

[B75] Elkoca E, Kantar F, Sahin F (2008). Influence of nitrogen fixing and phosphorus solubilizing bacteria on
the nodulation, plant growth, and yield of chickpea. J Plant Nut.

[B76] Elliott ET, Coleman DC (1988). Let the soil work for us. Ecol Bull.

[B77] Espinosa-Urgel M, Salido A, Ramos JL (2000). Genetic analysis of functions involved in adhesion of
*Pseudomonas putida* to seeds. J Bacteriol.

[B78] Estrada GA, Baldani VLD, Oliveira DM, Urquiaga S, Baldani JV (2013). Selection of phosphate-solubilizing diazotrophic
*Herbaspirillum* and *Burkholderia* strains and
their effect on rice crop yield and nutrient uptake. Plant Soil.

[B79] Ettema CH, Wardle DA (2002). Spatial soil ecology. Trends Ecol Evol.

[B80] Farajzadeh D, Yakhchali B, Aliasgharzad N, Sokhandan-Bashir N, Farajzadeh M (2012). Plant growth promoting characterization of indigenous
*Azotobacteria* isolated from soils in Iran. Curr Microbiol.

[B81] Farina RA, Beneduzi A, Ambrosini A, Campos SB, Lisboa BB, Wendisch V, Vargas LK, Passaglia LMP (2012). Diversity of plant growth-promoting rhizobacteria communities
associated with the stages of canola growth. Appl Soil Ecol.

[B82] Ferreira AS, Pires RR, Rabelo PG, Oliveira RC, Luz JMQ, Brito CH (2013). Implications of *Azospirillum brasilense* inoculation
and nutrient addition on maize in soils of the Brazilian Cerrado under greenhouse
and field conditions. Appl Soil Ecol.

[B83] Foley JA, DeFries R, Asner GP, Barford C, Bonan G, Carpenter SR, Chapin FS, Coe MT, Daily GC, Gibbs HK (2005). Global consequences of land use. Science.

[B84] Franche C, Lindström K, Elmerich C (2009). Nitrogen-fixing bacteria associated with leguminous and non-leguminous
plants. Plant Soil.

[B85] Frey M, Schullehner K, Dick R, Fiesselmann A, Gierl A (2009). Benzoxazinoid biosynthesis, a model for evolution of secondary
metabolic pathways in plants. Phytochemistry.

[B86] Fuqua C, Greenberg EP (2002). Listening in on bacteria: Acyl-homoserine lactone
signalling. Nat Rev Mol Cell Biol.

[B87] Gaiero JR, McCall CA, Thompson KA, Day NJ, Best AS, Dunfield KE (2013). Inside the root microbiome: Bacterial root endophytes and plant growth
promotion. Am J Bot.

[B88] Garcia de Salamone IE, Döbereiner J, Urquiaga S, Boddey RM (1996). Biological nitrogen fixation in *Azospirillum* strain
maize genotype associations as evaluated by ^15^N isotope dilution
technique. Biol Fertil Soils.

[B89] Ghimire R, Norton JB, Stahl PD, Norton U (2014). Soil microbial substrate properties and microbial community responses
under irrigated organic and reduced-tillage crop and forage production
systems. PLoS One.

[B90] Glass ADM (1989). Plant Nutrition: An Introduction to Current Concepts.

[B91] Glick BR (2005). Modulation of plant ethylene levels by the bacterial enzyme ACC
deaminase. FEMS Microbiol Lett.

[B92] Glick BR (2010). Using soil bacteria to facilitate phytoremediation. Biotechnol Adv.

[B93] Glick B (2012). Plant growth-promoting bacteria: Mechanisms and
applications. Scientifica.

[B94] Govindarajan M, Balandreau J, Muthukumarasamy R, Revathi G, Lakshminarasimhan C (2006). Improved yield of micropropagated sugarcane following inoculation by
endophytic *Burkholderia vietnamiensis*. Plant Soil.

[B95] Govindarajan M, Balandreau J, Kwon SW, Weon HY, Lakshminarasimhan C (2008). Effects of the inoculation of *Burkholderia
vietnamensis* and related endophytic diazotrophic bacteria on grain
yield of rice. Microb Ecol.

[B96] Granada C, Costa PB, Lisboa BB, Vargas LK, Passaglia LMP (2013). Comparison among bacterial communities present in arenized and
adjacent areas subjected to different soil management regimes. Plant Soil.

[B97] Gray EJ, Smith DL (2005). Intracellular and extracellular PGPR: Commonalities and distinctions
in the plant-bacterium signaling processes. Soil Biol Biochem.

[B98] Griffiths BS, Hallett PD, Kuan HL, Gregory AS, Watts CW, Whitmore AP (2008). Functional resilience of soil microbial communities depends on both
soil structure and microbial community composition. Biol Fertil Soils.

[B99] Griffiths BS, Philippot L (2012). Insights into the resistance and resilience of the soil microbial
community. FEMS Microbiol Rev.

[B100] Grover M, Ali SKZ, Sandhya V, Rasul A, Venkateswarlu B (2011). Role of microorganisms in adaptation of agriculture crops to abiotic
stresses. World J Microbiol Biotechnol.

[B101] Gulati HK, Chadha BS, Saini HS (2007). Production and characterization of thermostable alkaline phytase from
*Bacillus laevolacticus* isolated from rhizosphere
soil. J Ind Microbiol Biot.

[B102] Guttman D, McHardy AC, Schulze-Lefert P (2014). Microbial genome-enabled insights into plant-microorganism
interactions. Nat Rev Genet.

[B103] Haas D, Defago G (2005). Biological control of soil-borne pathogens by fluorescent
pseudomonads. Nat Rev Microbiol.

[B104] He K, Bauer CE (2014). Chemosensory signaling systems that control bacterial
survival. Trends Microbiol.

[B105] Holford ICR (1997). Soil phosphorus, its measurements and its uptake by
plants. Austr J Soil Res.

[B106] Hungria M, Campo RJ, Souza EM, Pedrosa FO (2010). Inoculation with selected strains of *Azospirillum
brasilense* and *A. lipoferum* improves yields of maize
and wheat in Brazil. Plant Soil.

[B107] Hungria M, Nogueira MA, Araujo RS (2013). Co-inoculation of soybeans and common beans with rhizobia and
azospirilla: Strategies to improve sustainability. Biol Fertil Soils.

[B108] Idriss EE, Makarewicz O, Farouk A, Rosner K, Greiner R, Bochow H, Richter T, Borriss R (2002). Extracellular phytase activity of *Bacillus
amyloliquefaciens* FZB45 contributes to its plant-growth-promoting
effect. Microbiol.

[B109] Ikeda AG, Bassani LL, Adamoski D, Stringari D, Cordeiro VK, Glienke C, Maria Steffens BR, Hungria M, Galli-Terasawa LV (2013). Morphological and genetic characterization of endophytic bacteria
isolated from roots of different maize genotypes. Microb Ecol.

[B110] Isawa T, Yasuda M, Awazaki H, Minamisawa K, Shinozaki S, Nakashita H (2010). Azospirillum sp strain B510 enhances rice growth and
yield.. Microbes Environ.

[B111] Jalili F, Khavazi K, Pazira E, Nejati A, Rahmani HA, Sadaghiani HR, Miransari M (2009). Isolation and characterization of ACC deaminase-producing fluorescent
pseudomonads, to alleviate salinity stress on canola (*Brassica
napus* L.) growth. J Plant Physiol.

[B112] James EK, Olivares FL, Baldani JI, Dobereiner J (1997). *Herbaspirillum*, an endophytic diazotroph colonizing vascular
tissue of *Sorghum bicolor* L. Moench. J Exp Bot.

[B113] Jensen JB, Egsgaard H, Onckelen HV, Jochimsen BU (1995). Catabolism of Indole-3-Acetic Acid and 4- and 5-Chloroindole-3-Acetic
Acid in *Bradyrhizobium japonicum*. J Bacteriol.

[B114] Jones DL (1998). Organic acids in the rhizosphere - A critical review. Plant Soil.

[B115] Jorquera MA, Hernández MT, Rengel Z, Marschner P, Mora ML (2008). Isolation of culturable phosphobacteria with both
phytate-mineralization and phosphate-solubilization activity from the rhizosphere
of plants grown in a volcanic soil. Biol Fertil Soils.

[B116] Jorquera MA, Crowley DE, Marschner P, Greiner R, Fernández MT, Romero D, Menezes-Blackburn D, De La Luz MM (2011). Identification of b-propeller phytase-encoding genes in culturable
*Paenibacillus* and *Bacillus* spp. from the
rhizosphere of pasture plants on volcanic soils. FEMS Microbiol Ecol.

[B117] Kabata-Pendias A (2004). Soil-plant transfer of trace elements-an environmental
issue. Geoderma.

[B118] Kerovuo J, Lauraeus M, Nurminen P, Kalkkinen N, Apajalahti J (1998). Isolation, characterization, molecular gene cloning, and sequencing of
a novel phytase from *Bacillus subtilis*. Appl Environ Microbiol.

[B119] Khalid A, Tahir S, Arshad M, Zahir ZA (2004). Relative efficiency of rhizobacteria for auxin biosynthesis in
rhizosphere and non-rhizosphere soils. Soil Res.

[B120] Khan MS, Zaidi A, Wani PA (2009). Role of phosphate-solubilizing microorganisms insustainable
agriculture - A review. Agron Sustain Dev.

[B121] Kibblewhite MG, Ritz K, Swift MJ (2008). Soil health in agricultural systems. Philos Trans R Soc Lond B Biol Sci.

[B122] Kobayashi T, Nishizawa NK (2012). Iron uptake, translocation, and regulation in higher
plants. Annu Rev Plant Biol.

[B123] Krewulak HD, Vogel HJ (2008). Structural biology of bacterial iron uptake. Biochim Biophys Acta.

[B124] Kumar V, Singh P, Jorquera MA, Sangwan P, Kumar P, Verma AK, Agrawal S (2013). Isolation of phytase-producing bacteria from Himalayan soils and their
effect on growth and phosphorus uptake of Indian mustard (*Brassica
juncea*). World J Microbiol Biotechnol.

[B125] Leigh JA, Coplin DL (1992). Exopolysaccharides in plant-bacterial interactions. Annu Rev Microbiol.

[B126] Lemanceau P, Bauer P, Kraemer S, Briat JF (2009). Iron dynamics in the rhizosphere as a case study for analyzing
interactions between soils, plants and microbes. Plant Soil.

[B127] Leveau JHJ, Lindow SE (2005). Utilization of the plant hormone indole-3-acetic acid for growth by
*Pseudomonas putida* strain 1290. Appl Environ Microbiol.

[B128] Loaces I, Ferrando L, Scavino AF (2011). Dynamics, diversity and function of endophytic siderophore-producing
bacteria in rice. Microb Ecol.

[B129] Long SR (2001). Genes and signals in the *Rhizobium* legume
symbiosis. Plant Physiol.

[B130] Lynch JM, Bragg E, Stewart BA (1985). Microorganisms and soil aggregate
stability. Advances in Soil Science.

[B131] Lynch JM (1990). The rhizosphere.

[B132] Majumder B, Kuzyakov Y (2010). Effect of fertilization on decomposition of ^14^C labelled
plant residues and their incorporation into soil aggregates. Soil Tillage Res.

[B133] Martinez JL, Sanchez MB, Martinez-Solano L, Hernandez A, Garmendia L, Fajardo A, Alvarez-Ortega C (2009). Functional role of bacterial multidrug efflux pumps in microbial
natural ecosystems. FEMS Microbiol Rev.

[B134] McLaughlin MJ, McBeath TM, Smernik R, Stacey SP, Ajiboye B, Guppy C (2011). The chemical nature of P accumulation in agricultural
soils-implications for fertilizer management and design: An Australian
perspective. Plant Soil.

[B135] Meneses CH, Rouws LF, Simões-Araújo JL, Vidal MS, Baldani JI (2011). Exopolysaccharide production is required for biofilm formation and
plant colonization by the nitrogen-fixing endophyte *Gluconacetobacter
diazotrophicus*. Mol Plant-Microbe Interact.

[B136] Miché L, Battistoni FJ, Gemmer S, Belghazi M, Reinhold-Hurek B (2006). Upregulation of jasmonate-inducible defense proteins and differential
colonization of roots of *Oryza sativa* cultivars with the
endophyte *Azoarcus* sp. Mol Plant-Microbe Interact.

[B137] Miller SH, Browne P, Prigent-Combaret C, Combes-Meynet E, Morrissey JP, O'Gara F (2010). Biochemical and genomic comparison of inorganic phosphate
solubilization in *Pseudomonas* species. Environ Microbiol Rep.

[B138] Moat AG, Foster JW, Moat AG, Foster JW (1995). Nitrogen metabolism. Microbial Physiology.

[B139] Monteiro RA, Schmidt MA, de Baura VA, Balsanelli E, Wassem R, Yates MG, Randi MAF, Pedrosa FO, de Souza EM (2008). Early colonization pattern of maize (*Zea mays* L.
Poales, Poaceae) roots by *Herbaspirillum seropedicae*
(Burkholderiales, Oxalobacteraceae). Genet Mol Biol.

[B140] Morel MA, Braña V, Castro-Sowinski S, Goyal Aakash (2012). Legume crops, importance and use of bacterial
inoculation to increase production. Plant Crop..

[B141] Mougel C, Offre P, Ranjard L, Corberand T, Gamalero E, Robin C, Lemanceau P (2006). Dynamic of the genetic structure of bacterial and fungal communities
at different developmental stages of *Medicargo truncatula gaertn*
cv jemalong line J5. New Phytol.

[B142] Muthukumarasamy R, Cleenwerk I, Revathi G, Vadivelu M, Janssens D, Hoste B, Gum KU, Park KD, Son CY, Sa T (2005). Natural association of *Gluconacetobacter
diazotrophicus* and diazotrophic *Acetobacter
peroxydans* with wetland rice. Syst Appl Microbiol.

[B143] Neal AL, Ahmad S, Gordon-Weeks R, Ton J (2012). Benzoxazinoids in root exudates of maize attract *Pseudomonas
putida* to the rhizosphere. PLoS One.

[B144] Neilands JB (1995). Siderophores: Structure and function of microbial iron transport
compounds. J Biol Chem.

[B145] Newton WE, Pedrosa FO, Hungria M, Yates MG, Newton WE (2000). Nitrogen fixation in perspective. Nitrogen Fixation: From Molecules to Crop Productivity.

[B146] Nicol D, Copaja SV, Wratten SD, Niemeyer HM (1992). A screen of worldwide wheat cultivars for hydroxamic acid levels and
aphid antixenosis. Ann Appl Biol.

[B147] Njoloma J, Tanaka K, Shimizu T, Nishiguchi T, Zakria M, Akashi R, Oota M, Akao S (2006). Infection and colonization of aseptically micropropagated sugarcane
seedlings by nitrogen-fixing endophytic bacterium, *Herbaspirillum*
sp. B501gfp1. Biol Fertil Soils.

[B148] Okon Y, Labandrera-Gonzalez CA (1994). Agronomic applications of *Azospirillum*: An evaluation
of 20 years of worldwide field inoculation. Soil Biol Biochem.

[B149] Oliveira ALM, Urquiaga S, Döbereiner J, Baldani JI (2002). The effect of inoculating endophytic N_2_ fixing bacteria on
micro propagated sugarcane plants. Plant Soil.

[B150] Onofre-Lemus J, Hernández-Lucas I, Girard L, Caballero-Mellado J (2009). ACC (1-aminocyclopropane-1-carboxylate) deaminase activity, a
widespread trait in *Burkholderia* species, and its
growth-promoting effect on tomato plants. App Environ Microbiol.

[B151] Pedrosa FO, Monteiro RA, Wassem R, Cruz LM, Ayub RA, Colauto NB, Fernandez MA, Fungaro MHP, Grisard EG, Hungria M (2011). Genome of *Herbaspirillum seropedicae* strain SmR1, a
specialized diazotrophic endophyte of tropical grasses. PLoS Genet.

[B152] Prinsen E, Costacurta A, Michiels K, Vanderleyden J, Onckelen H (1993). *Azospirillum brasilense* indole-3-acetic acid biosynthesis:
Evidence for a non-tryptophan dependent pathway. Mol Plant-Microbe Interact.

[B153] Pulleman M, Creamer R, Hamer U, Helder J, Pelosi C, Peres G, Rutgers M (2012). Soil biodiversity, biological indicators and soil ecosystem
services-an overview of European approaches. Curr Opin Environ Sustain.

[B154] Qin L, Jiang H, Tian J, Zhao J, Liao H (2011). Rhizobia enhance acquisition of phosphorus from different sources by
soybean plants. Plant Soil.

[B155] Ramachandran VK, East AK, Karunakaran R, Downie JA, Poole PS (2011). Adaptation of *Rhizobium leguminosarum* to pea, alfalfa
and sugar beet rhizospheres investigated by comparative
transcriptomics. Genome Biol.

[B156] Remans R, Beebe S, Blair M, Manrique G, Tovar E, Rao I, Croonenborghs A, Torres-Gutierrez R, El-Howeity M, Michiels J (2008). Physiological and genetic analysis of root responsiveness to
auxin-producing plant growth-promoting bacteria in common bean (*Phaseolus
vulgaris* L.). Plant Soil.

[B157] Richardson AE, Simpson RJ (2011). Soil microorganisms mediating phosphorus availability. Plant Physiol.

[B158] Robinson CJ, Bohannan BJ, Young VB (2010). From structure to function: The ecology of host-associated microbial
communities. Microbiol Mol Biol Rev.

[B159] Rocha FR, Papini-Terzi FS, Nishiyama MY, Vêncio RZN, Vicentini R, Duarte RDC, de Rosa VE, Vinagre F, Barsalobres C, Medeiros AM (2007). Signal transduction-related responses to phytohormones and
environmental challenges in sugarcane. BMC Genomics.

[B160] Rodríguez H, Fraga R (1999). Phosphate solubilizing bacteria and their role in plant growth
promotion. Biotechnol Adv.

[B161] Rodríguez H, Fraga R, Gonzalez T, Bashan Y (2006). Genetics of phosphate solubilization and its potential applications
for improving plant growth-promoting bacteria. Plant Soil.

[B162] Rodriguez-Navarro DN, Dardanelli MS, Ruiz-Sainz JE (2007). Attachment of bacteria to the roots of higher plants. FEMS Microbiol Lett.

[B163] Rosenthal GA (1972). Investigations of canavanine biochemistry in the jack bean plant,
*Canavalia ensiformis* (L) DC: II Canavanine biosynthesis in the
developing plant. Plant Physiol.

[B164] Rothballer M, Eckert B, Schmid M, Fekete A, Schloter M, Lehner A, Pollmann S, Hartmann A (2008). Endophytic root colonization of gramineous plants by
*Herbaspirillum frisingense*. FEMS Microbiol Ecol.

[B165] Rovira AD (1965). Interactions between plant roots and soil
micro-organisms. Annu Rev Microbiol.

[B166] Ruamps LS, Nunan N, Chenu C (2011). Microbial biogeography at the soil pore scale. Soil Biol Biochem.

[B167] Rudrappa T, Czymmek KJ, Paré PW, Bais HP (2008). Root-secreted malic acid recruits beneficial soil
bacteria. Plant Physiol.

[B168] Saleem M, Arshad M, Hussain S, Bhatti AS (2007). Perspective of plant growth promoting rhizobacteria (PGPR) containing
ACC deaminase in stress agriculture. J Ind Microbiol Biotechnol.

[B169] Schenk ST, Stein E, Kogel KH, Schikora A (2012). Arabidopsis growth and defense are modulated by bacterial quorum
sensing molecules. Plant Signal Behav.

[B170] Schmidt MW, Torn MS, Abiven S, Dittmar T, Guggenberger G, Janssens IA, Kleber M, Kögel-Knabner I, Lehmann J, Manning DAC (2011). Persistence of soil organic matter as an ecosystem
property. Nature.

[B171] Sevilla M, Burris RH, Gunapala N, Kennedy C (2001). Comparison of benefit to sugarcane plant growth and
^15^N_2_ incorporation following inoculation of sterile
plants with *Acetobacter diazotrophicus* wild-type and
Nif^−^ mutant strains. Mol Plant-Microbe Interact.

[B172] Shade A, Peter H, Allison SD, Baho L, Berga M, Bürgmann H, Huber DH, Langenheder S, Lennon JT, Martiny JBH (2012). Fundamentals of microbial community resistance and
resilience. Front Microbiol.

[B173] Shaharoona B, Arshad M, Zahir ZA (2006). Effect of plant growth promoting rhizobacteria containing
ACC-deaminase on maize (*Zea mays* L.) growth under axenic
conditions and on nodulation in mung bean (*Vigna radiata*
L.). Lett Appl Microbiol.

[B174] Sharma SB, Sayyed RZ, Trivedi MH, Gobi TA (2013). Phosphate solubilizing microbes: Sustainable approach for managing
phosphorus deficiency in agricultural soils. Springerplus.

[B175] Sharma SK, Ramesh A, Johri BN (2013). Isolation and characterization of plant growth promoting
*Bacillus amyloliquefaciens* strain sks_bnj_1 and its influence
on rhizosphere soil properties and nutrition of soybean (*Glycine
max* L. Merrill). J Virol Microbiol.

[B176] Shenoy VV, Kalagudi GM, Gurudatta BV (2001). Towards nitrogen autotrophic rice. Curr Sci.

[B177] Singh P, Kumar V, Agrawal S (2014). Evaluation of phytase producing bacteria for their plant growth
promoting activities. Int J Microbiol.

[B178] Shridhar BS (2012). RNitrogen fixing microorganisms. Int J Microbiol Res.

[B179] Six J, Paustian K, Elliott ET, Combrink C (2000). Soil structure and organic matter. I. Distribution of aggregate-size classes and
aggregate-associated carbon. Soil Sci Soc Am J.

[B180] Souza R, Beneduzi A, Ambrosini A, Costa PB, Meyer J, Vargas LK, Schoenfeld R, Passaglia LMP (2013). The effect of plant growth-promoting rhizobacteria on the growth of
rice (*Oryza sativa* L.) cropped in southern Brazilian
fields. Plant Soil.

[B181] Souza R, Meyer J, Schoenfeld R, Costa PB, Passaglia LMP (2014). Characterization of plant growth-promoting bacteria associated with
rice cropped in iron-stressed soils. Ann Microbiol.

[B182] Spaepen S, Vanderleyden J, Remans R (2007). Indole-3-acetic acid in microbial and microorganism-plant
signaling. FEMS Microbiol Rev.

[B183] Spaepen S, Dobbelaere S, Croonenborghs A, Vanderleyden J (2008). Effects of *Azospirillum brasilense* indole-3-acetic
acid production on inoculated wheat plants. Plant Soil.

[B184] Stein RJ, Duarte GL, Spohr MG, Lopes SIG, Fett JP (2009). Distinct physiological responses of two rice cultivars subjected to
iron toxicity under field conditions. Ann Appl Biol.

[B185] Straub D, Yang H, Liu Y, Tsap T, Ludewig U (2013). Root ethylene signalling is involved in *Miscanthus
sinensis* growth promotion by the bacterial endophyte
*Herbaspirillum frisingense* GSF30T. J Exp Bot.

[B186] Sun Y, Cheng Z, Glick BR (2009). The presence of a 1-aminocyclopropane-1-carboxylate (ACC) deaminase
deletion mutation alters the physiology of the endophytic plant growth-promoting
bacterium *Burkholderia phytofirmans* PsJN. FEMS Microbiol Lett.

[B187] Szilagyi-Zecchin VJ, Ikeda AC, Hungria M, Adamoski D, Kava-Cordeiro V, Glienke C, Galli-Terasawa LV (2014). Identification and characterization of endophytic bacteria from corn
(*Zea mays* L.) roots with biotechnological potential in
agriculture. AMB Express.

[B188] Terakado-Tonooka J, Ohwaki Y, Yamakawa H, Tanaka F, Yoneyama T, Fujihara S (2008). Expresses *nifH* genes of endophytic bacteria detected
in field-growth sweet potatoes (*Ipomoea batata*
L.). Microbes Environl.

[B189] Thaweenut N, Hachisuka Y, Ando S, Yanagisawa S, Yoneyama T (2011). Two seasons' study on *nifH* gene expression and
nitrogen fixation by diazotrophic endophytes in sugarcane
(*Saccharum* spp. hybrids): Expression of *nifH*
genes similar to those of rhizobia. Plant Soil.

[B190] Torres AR, Kaschuk G, Saridakis GP, Hungria M (2012). Genetic variability in *Bradyrhizobium japonicum*
strains nodulating soybean *Glycine max* (L.)
Merrill. World J Microbiol Biotechnol.

[B191] Tortora ML, Díaz-Ricci JC, Pedraza RO (2011). Azospirillum brasilense siderophores with antifungal activity against
Colletotrichum acutatum. Arch Microbiol.

[B192] Upadhyay SK, Singh JS, Saxena AK, Singh DP (2012). Impact of PGPR inoculation on growth and antioxidant status of wheat
under saline conditions. Plant Biol.

[B193] Valverde A, Burgos A, Fiscella T, Rivas R, Velázquez E, Rodríguez-Barrueco C, Cervantes E, Chamber M, Igual J-M (2006). Differential effects of coinoculations with *Pseudomonas
jessenii* PS06 (a phosphate-solubilizing bacterium) and
*Mesorhizobium ciceri* C-2/2 strains on the growth and seed
yield of chickpea under greenhouse and field conditions. Plant Soil.

[B194] Vogel C, Babin D, Pronk GJ, Heister K, Smalla K, Kögel-Knabner I (2014). Establishment of macro-aggregates and organic matter turnover by
microbial communities in long-term incubated artificial soils. Soil Biol Biochem.

[B195] Vos M, Wolf AB, Jennings SJ, Kowalchuk GA (2013). Micro-scale determinants of bacterial diversity in
soil. FEMS Microbiol Rev.

[B196] Walker V, Bertrand C, Bellvert F, Moënne-Loccoz Y, Bally R, Comte G (2011a). Host plant secondary metabolite profiling shows a complex,
strain-dependent response of maize to plant growth-promoting rhizobacteria of the
genus *Azospirillum*. New Phytol.

[B197] Walker V, Couillerot O, Von Felten A, Bellvert F, Jansa J, Maurhofer M, Bally R, Moënne-Loccoz Y, Comte G (2011b). Variation of secondary metabolite levels in maize seedling roots
induced by inoculation with *Azospirillum, Pseudomonas* and
*Glomus* consortium under field conditions. Plant Soil.

[B198] Wartiainen I, Eriksson T, Zheng W, Rasmussen U (2008). Variation in the active diazotrophic community in rice
paddy-*nifH* PCR-DGGE analysis of rhizosphere and bulk
soil. Appl Soil Ecol.

[B199] Wasternack C (2007). Jasmonates: An update on biosynthesis, signal transduction and action
in plant stress response, growth and development. Ann Bot.

[B200] Willems A, Velazquez E, Rodryguez-Barrueco C (2007). The taxonomy of rhizobia: An overview. First International Meeting on Microbial Phosphate
Solubilization.

[B201] Wolf AB, Vos M, de Boer W, Kowalchuk GA (2013). Impact of matric potential and pore size distribution on growth
dynamics of filamentous and non-filamentous soil bacteria. PLoS One.

[B202] Yoon SJ, Choi YJ, Min HK, Cho KK, Kim JW, Lee SC, Jung YH (1996). Isolation and identification of phytase producing bacterium,
*Enterobacter* sp 4, and enzymatic properties of phytase
enzyme.. Enzyme Microb Tech.

[B203] You M, Nishiguchi T, Saito A, Isawa T, Mitsui H, Minamisawa K (2005). Expression of the *nifH* gene of a
*Herbaspirillum* endophyte in wild rice species: Daily rhythm
during the light-dark cycle. Appl Environ Microbiol.

[B204] Young IM, Ritz K (1999). Tillage, habitat space and function of soil microbes. Soil Tillage Re.

[B205] Zahir ZA, Munir A, Asghar HN, Shaharoona B, Arshad M (2008). Effectiveness of rhizobacteria containing ACC deaminase for growth
promotion of peas (*Pisum sativum*) under drought
conditions. J Microbiol Biotechnol.

[B206] Zehr JP, Jenkins BD, Short SM, Steward GF (2003). Nitrogenase gene diversity and microbial community structure: A
cross-system comparison. Environ Microbiol.

[B207] Zhang H (1994). Organic matter incorporation affects mechanical properties of soil
aggregates. Soil Tillage Res.

[B208] Zhang J, Boone L, Kocz R, Zhang C, Binns AN, Lynn DG (2000). At the maize/*Agrobacterium* interface: Natural factors
limiting host transformation. Chem Biol.

[B209] Zhang S, Li Q, Lü Y, Zhang X, Liang W (2013). Contributions of soil biota to C sequestration varied with aggregate
fractions under different tillage systems. Soil Biol Biochem.

